# Spatio-structural granularity of biological material entities

**DOI:** 10.1186/1471-2105-11-289

**Published:** 2010-05-28

**Authors:** Lars Vogt

**Affiliations:** 1 Institut für Evolutionsbiologie und Ökologie, Universität Bonn; An der Immenburg 1, D-53121 Bonn, Germany

## Abstract

**Background:**

With the continuously increasing demands on knowledge- and data-management that databases have to meet, ontologies and the theories of granularity they use become more and more important. Unfortunately, currently used theories and schemes of granularity unnecessarily limit the performance of ontologies due to two shortcomings: (i) they do not allow the integration of multiple granularity perspectives into one granularity framework; (ii) they are not applicable to cumulative-constitutively organized material entities, which cover most of the biomedical material entities.

**Results:**

The above mentioned shortcomings are responsible for the major inconsistencies in currently used spatio-structural granularity schemes. By using the Basic Formal Ontology (BFO) as a top-level ontology and Keet's general theory of granularity, a granularity framework is presented that is applicable to cumulative-constitutively organized material entities. It provides a scheme for granulating complex material entities into their constitutive and regional parts by integrating various compositional and spatial granularity perspectives. Within a scale dependent resolution perspective, it even allows distinguishing different types of representations of the same material entity. Within other scale dependent perspectives, which are based on specific types of measurements (e.g. weight, volume, etc.), the possibility of organizing instances of material entities independent of their parthood relations and only according to increasing measures is provided as well. All granularity perspectives are connected to one another through overcrossing granularity levels, together forming an integrated whole that uses the *compositional object perspective *as an integrating backbone. This granularity framework allows to consistently assign structural granularity values to all different types of material entities.

**Conclusions:**

The here presented framework provides a spatio-structural granularity framework for all domain reference ontologies that model cumulative-constitutively organized material entities. With its multi-perspectives approach it allows querying an ontology stored in a database at one's own desired different levels of detail: The contents of a database can be organized according to diverse granularity perspectives, which in their turn provide different *views *on its content (i.e. data, knowledge), each organized into different levels of detail.

## 1 Background

Due to the ever increasing amounts of data generated in biomedical research, data bases for managing these data become more important. And due to the need for more standardization in form of formalization and externalization (see [1,2]) that accompanies this trend, bioinformatics and ontology development received more and more attention within biomedical sciences (e.g. [3-12]).

Biology and medicine belong to the major domains of application of ontologies. The large amounts of different kinds of biomedical data and the organizational complexity of organisms thereby often require bio-ontologies to provide formal tools for distinguishing data types and for managing different levels of organizational complexity. While ontologies accomplish the former by organizing their terms and concepts along the lines of is-a relations that result in an encaptic (i.e. nested) hierarchical organization of terms and concepts, they usually accomplish the latter by differentiating *levels of granularity *along the lines of part-whole relations resulting in granularity trees (e.g. [13,14]).

When modeling their domain of reality and representing different types of biological material entities, and their properties and relations, *biologists *commonly refer to two distinct ways of hierarchically ordering them. On the one hand, biologists use *taxonomic hierarchies *in order to structure all knowledge referring to different types of organisms, like the Linnaean zoological taxonomy of species, genera, families and orders [15], resulting in a more or less universal referencing system of biological knowledge. On the other hand, biologists also use *partonomic hierarchies*, which organize biological objects according to their part-whole relations. These hierarchies resemble granularity trees for biological material entities. Several of such rather informal hierarchical systems have been proposed in the past. Some, like Eldredge's *somatic hierarchy *[16] (see also [17,18]) are focused on biological anatomical objects, and usually include *subatomic particle *<*atom *<*molecule *<*organelle *<*cell *<*tissue *<*organ *<*organ system *<*individual organism *as their typical ranks/levels (here and in the following ' < ' is used to indicate *lower-level-than *relationships). Other hierarchies are focused on typical material entities of other biological disciplines. Ecological hierarchies (e.g. [18]), for instance, usually include *individual *<*population *<*community *<*biotic province *<*biosphere*. Some hierarchies are focused on genetic units (genetic hierarchy; see [19]), genealogical units (*genealogical hierarchy *[20]), or evolutionary units (homology hierarchy [21]; phylogenetic trees/cladograms).

Thus, conceptualizing organisms and their parts along the lines of part-whole relations does not represent a new idea and has been done before in biology. It results in a hierarchical system of anatomical structures and their respective terms and concepts that is usually referred to in the biological literature as *levels of (biological) organization *or *levels of complexity *[22,23] (see also *scalar hierarchy *[24,25]; *cumulative constitutive hierarchy *[19]; *building block systems *[26]; *Theorie des Schichtenbaus der Welt *[27]). However, these rather informal approaches of biologists are insufficient today, since they cannot be directly utilized in databases and fail to suffice modern standards of data management. Therefore, and considering the organizational complexity of most biological material entities, an adequate theory of granularity is required that is unambiguously applicable to the biomedical domain. A central question in this respect is whether the currently applied theories of granularity really rest on realistic assumptions about the spatio-structural organization of the material entities that belong to the biological domain of reality, or not. Which types of hierarchical systems do biologists use, and can they be mapped onto the commonly used granularity schemes of, for example, zoological anatomy?

Theories of granularity are also relevant for the development of a desperately required overarching information framework [28] promoting the integration and exchange of different types of biomedical data. Many currently highly anticipated scientific questions, like those involved in multidisciplinary studies of gene-phene interaction or neuroscience, have to integrate very diverse types of data and have to exchange data between different databases and between scientists with different scientific backgrounds. Thereby, they often have to deal with vast amounts of various different types of data generated in biomedical sciences today. Such integration and exchange of data does not only involve standardization of data and metadata [2,29,30], but requires an additional organization of data and knowledge within databases that surpasses the organization that databases usually bring about, which is one of the key objectives of a general granularity framework.

Current ontologies in databases use granularity frameworks that merely allow them to store knowledge and data about their subject domain, browse and manually annotate data, but cannot properly be used for data integration and inferencing. This is partly due to constraints that result from a granularity framework that limits the organization of knowledge and data to a single granularity perspective. It may be adequate for many *terminology-based application ontologies *[14,31], which only aim to provide a system of terms (i.e. controlled vocabularies) that has been built to meet very specific purposes and needs. However, this limitation becomes very problematic for *domain reference ontologies *[14,31], since they represent general-purpose resources that are developed to support a range of very different interests and purposes. Organizing knowledge and data with such domain reference ontologies on the basis of a single-perspective granularity framework while satisfying these different interests and purposes is not possible.

In the following, commonly applied granularity theories will be briefly described and subsequently exposed to a reality check against a specific type of organization of material entities that typically, but not exclusively, can be found in the domain of biology. After discussing the differences between a granularity of *instances *(i.e. particulars; e.g. a particular individual cell) and a granularity of *types *(i.e. universals; e.g. a specific type of cell) with respect to the biological type of organization, a general integrated spatio-structural granularity framework will be presented and discussed that is based on Keet's general theory of granularity [32]. It accommodates different spatial, compositional, and even scale dependent granularity perspectives that, together, enable the exhaustive representation of all spatio-structural aspects of material entities of the biological type of organization.

## 2 Granularity and Different Types of Hierarchies

### 2.1 Granular Partition and Granularity Tree

In mathematics and logic a ***partial order ***is a binary relation *P *that is *transitive *(if *x *has relation *P *to *y *and *y *has relation *P *to *z*, than *x *has relation *P *to *z*: (Pxy)(Pyz) → Pxz), *reflexive *(*x *has relation *P *to itself: Pxx), and *antisymmetric *(if *x *has relation *P *to *y *and *y *has relation *P *to *x*, than *x *and *y *are identical: ((Pxy)(Pyx) → x = y).

***Granular partitions***, which represent a key concept for theories of granularity for knowledge representation and ontology design, are based on partial ordering relations [33-36]. Granular partitions are involved in all listing, sorting, cataloging, and mapping activities and represent hierarchical partitions consisting of cells and subcells, with the latter being contained within the former (with the term 'cell' used here in its general, non-biological meaning). Granular partitions require a theory of the relations between cells and subcells of a given partition that satisfies the following conditions: (i) the subcell relation has to be transitive, reflexive, and antisymmetric (i.e. a partial ordering relation), (ii) the existence of a root cell (i.e. a unique maximal cell) of which all subcells are parts, (iii) chains of nested cells are of finite length, and (iv) if two cells overlap, then one is a subcell of the other, which excludes partial overlap [33-36]. Additionally, granular partitions require a theory of the relations between cells and entities in reality (i.e. projective relation to reality [33-35]).

Granular partitions are granular insofar as they can recognize a material entity without having to recognize all its parts (for a more general approach to granularity see *3.2 Keet's General Formal Theory of Granularity*). And since every finite partition can be represented as a rooted tree of finite depths [33,35,37,38] (i.e. a rooted directed graph without cycles [39]), granular partitions can be represented as ***granularity trees ***[36].

Within a granularity tree different levels of granularity can be distinguished, with a level being a *cut *(sensu [40]) in the tree structure [37]. The root of a granularity tree represents a cut itself and therewith a level of granularity, and all immediate children of the root another cut/level. The elements that form a level of granularity are *pair-wise disjoint *(i.e. no entity instantiates two different types of the same level of granularity). Levels of granularity are *exhaustive *in the sense that for every entity in the domain of interest of an ontology there exists some other entity in the same domain which belongs to another level of granularity and the former entity stands in a partial ordering relation to the latter, or vice versa [36]. Moreover, if the partitioning relation is mereological (e.g., part-whole relation), the parts exhaustively sum to the whole in a granularity tree, i.e. all entities belonging to one level of granularity form parts of entities of the next higher level of granularity [36].

Reitsma and Bittner [36] distinguish two types of granularity trees: (i) ***bona fide granularity trees ***with all entities being bona fide (i.e., entities that exist independently of human partitioning activities - e.g. objects that possess only physiologically discontinuous boundaries) and (ii) ***fiat granularity trees ***with all entities being fiat (i.e., entities that are created by human partitioning activities - e.g. entities that possess a physiologically continuous and therefore somewhat arbitrary boundary to a neighboring entities).

### 2.2 Reality Check: Cumulative Constitutive Hierarchies

**Figure 1:**
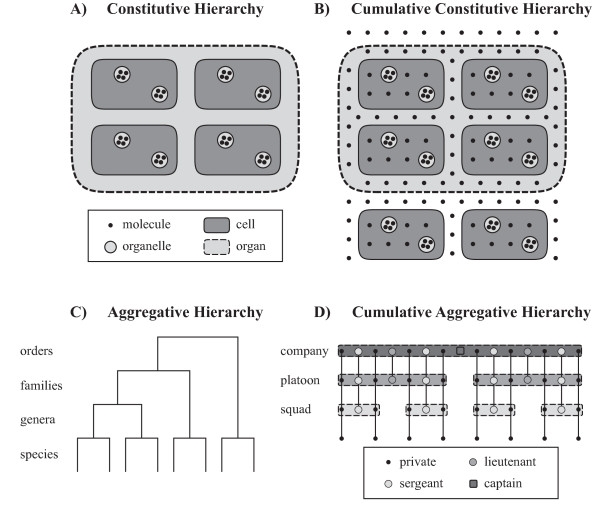
**Four different Types of Hierarchies used in Biology**. **A) **A *constitutive hierarchy *of molecules, organelles, cells, and organs of a multicellular organism, in which all molecules are contained in organelles, all organelles in cells, and all cells in organs. **B) **The more realistic case of a *cumulative constitutive hierarchy *of molecules, organelles, cells and organs of a multicellular organism, in which not all molecules are contained in organelles or cells, and not all cells in organs. **C) **An *aggregative hierarchy *of species being aggregated to genera, genera to families and families to orders, just like in a traditional Linnaean taxonomy. **D) **A *cumulative aggregative hierarchy*, as it can be found in the hierarchical organization of military stuff, in which individuals with higher ranks (i.e. sergeants, lieutenants, captains) 'emerge' in aggregates of higher order (i.e. squads, platoons, companies).

By characterizing hierarchies as being based on *strict partial ordering *relations (*transitive*, *antisymmetric*, *irreflexive *(*x *cannot have relation *P *to itself: ¬P*xx*); e.g. [41-43]), some biologists [19,43,44] have differentiated four basic types of hierarchical systems (see Fig. 1). The stronger irreflexive binary relation of strict partial orderings can be defined in terms of the respective weaker reflexive relation of partial ordering (i.e. partial orderings represent the more general case [45]). A strict partial ordering is the reflexive reduction of its corresponding partial ordering. Therefore, the following types of hierarchies can be discussed within the framework of partial ordering, granular partitions, and granularity trees provided above.

1) ***Constitutive hierarchy ***[46]: *"(...) the units at each level are physical parts of the collectives at the next higher level, where they do not have independent existences, and this situation continues up the level of inclusiveness" *([19], p. 25). In other words, higher level entities consist of physically joined elements, like cells in a multicellular organism [43,47]. Constitutive hierarchies are based on mereological (i.e., partonomic) inclusion that results from a proper part-whole relation (i.e. *irreflexive *part-whole relation) as the ordering relation. Moreover, since all objects belonging to one level of granularity form parts of objects of the next higher level of granularity, parts exhaustively sum to the whole and thus represent mereological granularity trees [36] (see Fig. 1A).

2) ***Aggregative hierarchy ***[46]: *"(...) the basic units are physically independent and remain so, but are organized into collectives that become units at the next higher level, and these in turn are organized into collectives at a succeeding level, and so forth in ascending degrees of inclusiveness" *([19], p. 25). Put differently, higher level entities consist of elements that are not physically connected, but only associated with each other, like organisms in a population [43]. Depending on what one understands as 'associated', aggregative hierarchies can either be based on mereological/meronymic inclusion that results from a very general notion of (proper) part-whole relationships (e.g. ecological hierarchies [18,19]), or on taxonomic inclusion [48] that results from is-a relations (e.g. Linnaean taxonomic hierarchy;). In case of the former, they represent mereological granularity trees (see Fig. 1C).

3-4) ***Cumulative hierarchy ***(see also *somatic hierarchy *[16]): In a cumulative hierarchy, elements of levels below the next lower level contribute to a certain level in the hierarchy. Many multicellular organisms exhibit this type of hierarchical organization, as for instance humans, in which extracellularly located bone matrix and blood plasma (both not consisting of cells) together with tissues and organs form the whole organism [19,43,47]. In analogy to the two types of hierarchies discussed above, Valentine and May [19] distinguish ***cumulative aggregative hierarchies ***from ***cumulative constitutive hierarchies***. An example for the former is the organization of military stuff, with privates in the lowest level, squads, consisting of privates and sergeants, in the next level, platoons, consisting of privates, sergeants, and lieutenants, in the following level, and companies, consisting of privates, sergeants, lieutenants, and captains, in the highest level (see Fig. 1D). An example for a cumulative constitutive hierarchy is the organization of extracellular matrix, i.e. ECM, in a multicellular metazoan organism. ECM is a macromolecular formation located outside of cells. It is not a component of cells, but it is a component of tissues and therefore also of organs, organ systems, and organisms (see Fig. 1B). Therefore, in most multicellular organisms, not all macromolecules are organized in cells, since their cells are embedded in ECM. The same applies to some fluids, which may be thought of as components of organs but not of the tissues of these organs. In other words, in general organs are made up of tissue, tissue of cells, and cells of (macro-) molecules, but a (macro-) molecule is not necessarily part of a cell, a cell not necessarily part of tissue, and tissue not necessarily part of an organ [49].

This situation is not unique to biology. Cumulative-constitutively organized material entities can be found in many other domains as well. After all, not all protons and neurons are organized within atoms and not all atoms within molecules, which shows that the physical domain is also organized in a cumulative constitutive way.

## 3 Granularity of Instances and of Types and the Problems with Cumulative Constitutive Hierarchies

**Figure 2:**
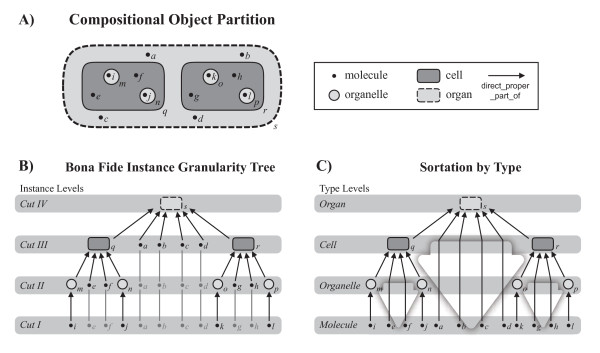
**Granularity of Instance vs Granularity of Types**. **A) **Compositional object partitions of an organ. Three partitions are shown: (i) into cells (q, r) and extracellular molecules (a-d); (ii) into organelles (m-p) and cellular molecules (a-h); and (iii) into organelle molecules (a-l). **B) **The bona fide granularity tree based on these three partitions. The organ itself and each of its partitions represent a cut in the tree and thus an instance granularity level. **C) **The extension of 'molecule' (i.e. the distribution of instances of 'molecule') of the organ crosses the boundaries of instance granularity levels. Therefore, the instance granularity tree cannot be directly transformed into or mapped upon a type granularity tree. But by following the simple and intuitive rule that a type occupies the lowest granularity level of its instances, one can nevertheless infer granularity levels for types.

Common approaches to granularity, such as the granular partitions and granularity tree approach discussed above, but also Kumar et al.'s approach [13] (which will be discussed in detail further below), all have problems with cumulative constitutively organized entities. When looking at the granularity of a particular entity and its particular parts, and thus when modeling the granularity of instances and not of types, the granulation of cumulative-constitutively organized entities causes only minor problems. One can, for example, partition an organ into its direct proper parts, which are cells and the molecules surrounding the cells (Fig. 2A). These parts all belong to the same cut in the corresponding granularity tree and thus to the same instance granularity level, although they instantiate different basic types. In a next step one can partition the cells into their organelles and their cellular molecules surrounding these organelles. These parts all belong to another cut in the tree and thus to the adjacently lower instance granularity level. As a last step, one can partition the organelles into their molecules, which provides the lowest instance granularity level (Fig. 2B). All this can be done in line with the granular partitions and granularity tree approach discussed above. The only drawback with cumulative-constitutively organized entities, opposed to constitutively organized entities, is that the mereological sum of all entities belonging to one instance granularity level does not always sum to the whole entity (which violates Kumar et al.'s third principle [13]; see further below). This would only hold, if some particular molecules would belong to more than one level, which would, however, violate the non-partially-overlap principle.

Whereas modeling cumulative-constitutively organized entities causes only minor problems when dealing with instances, the situation changes fundamentally when dealing with types. Ontologies usually deal with types and not with instances. A granularity framework for ontologies therefore has to deal with types, and it is the granularity of types that causes problems. Due to the cumulative constitutive organization, different instances of the same type can belong to different granularity levels. In the example above, some particular molecules together with some particular cells belong to the granularity level directly below the 'organ' level, whereas other particular molecules together with some particular organelles belong to the next lower level of granularity, and still other particular molecules belong to the lowest level of granularity (Fig. 2B). In other words, in cumulative-constitutively organized entities the extensions of some types (here it is 'molecule') cross the boundary between different levels of instance granularity. As a consequence, one cannot directly transform or map the topology of an instance granularity tree to its corresponding type granularity tree, since the resulting tree would violate granular partitions condition (iv) (i.e. *if two cells overlap, then one is a subcell of the other, excluding partial overlap*).

The question is, how one can deal with this situation in terms of a granularity of types. The intuitive solution, which is also reflected in the term 'cumulative constitutive hierarchy', is to rank types according to the lowest level of granularity of their instances. This sortation-by-type functions like a granular sedimentation of all the instances of one type to the lowest level they occupy (Fig. 2C). While this represents a pragmatic solution to the question of how to infer type granularity, it poses significant problems for the common approaches to granularity. All the parts of a cumulative-constitutively organized material entity that are objects belonging to the same particular type granularity level do not exhaustively sum to their respective wholes - *not all *objects belonging to one level of type granularity form parts of the next higher level of type granularity. This either violates one criterion of Reitsma and Bittner's [36] mereological bona fide granularity trees, or the tree contains only two levels (i.e. an atom-molecule level and a complex-whole level - for the example from above, no 'organelle' and no 'cell' level). The alternative would be that cumulative-constitutively organized material entities could only be partitioned forming fiat granularity trees, which in case of biological anatomy would represent a conceptualization of structural organization that contradicts all common research practice in biology. Thus, since the part-whole relations found in metazoan organisms are of the cumulative type [47], cumulative constitutive organization represents the reality that all attempts of developing formally stringent representations of levels of type granularity of biomedical material entities have to cope and to comply with. All partitioning schemes that do not comply to this fact are of limited applicability. This holds particularly for all comparative studies that involve data from specimens of different species or taxa.

Another problem with biological entities and their organization within type granularity trees results from their tremendous diversity. The claim that objects belonging to the same level of type granularity should have roughly the same size is not realistic. This becomes obvious when considering the impressive variability of real organisms, their mutability during development, their modification of scale and proportions during growth, their flexibility in scales and proportions under varying environmental conditions, and their evolutionary diversity of phenotypes across species. Already the size of cells within one organism usually vary considerably (e.g. oocytes, sperms, giant axons). Ear bones of some whale species are about 100 times smaller than their ribs. Some *Caulerpa *algae may grow to 3 m in length, whereas some *Mycoplasma *bacteria have a diameter of only 10 μm.

## 4 Theories of Granularity

Schemes of type granularity, although mostly not accompanied by an explicit presentation of formally defined levels of granularity, can be found in many bio-ontologies, like those that can be found via the BioPortal web-service of the National Center for Biomedical Ontology [50]. Although formally more stringent than the schemes proposed by most biologists, the actual granularity schemes of ontologies suggested so far show a considerable amount of variety (see Table 1). This variety cannot be explained solely by their differences in scope and domain of application (i.e., taxonomic coverage and coverage of granularity levels - some ontologies are restricted to a specific taxon and/or a set of specific levels of granularity). This implies that the theories of granularity applied (if any) obviously differ fundamentally from one another. No commonly accepted approach to type granularity has been agreed upon. Thus, despite its importance, how to properly organize anatomy ontologies (particularly cross-species anatomy ontologies) and define and demarcate different levels of type granularity is obviously still an open question.

**Table 1:** Granularity Schemes used in Ontologies of Anatomy.

Levels of Granularity [ordered from fine < coarse]	Domain/Application	Parent Class Affiliation	Ontology/Controlled Vocabulary	Version
'macromolecular complex' < 'organelle part' < 'organelle' < 'cell part' < 'cell'; *additional types: *"envelope', 'extracellular region', 'extracellular region part', 'membrane-enclosed lumen', 'symplast', 'synapse', 'synapse part', 'virion', 'virion part'	genomic and proteomic	'cellular component'	**Gene Ontology (GO)**	1.689

**units of structural organization: **'Biological macromolecule' < 'Cell' < 'Portion of tissue' < 'Organ' < 'Organ system' < 'Cardinal body part' < 'Body'; **subdivisions/cardinal parts of units: **'Cardinal cell part', 'Cardinal tissue part', 'Cardinal organ part', 'Organ system subdivision', 'Subdivision of cardinal body part'; **'cross-granular' types, spanning over several levels of granularity: **'Acellular anatomical structure', 'Anatomical cluster', 'Gestational structure', 'Vestigial embryonic structure'	human anatomy - to a limited degree also vertebrate anatomy	'Anatomical structure'	**Foundational Model of Anatomy (FMA)**	3.0

*MicroscopicStructure, in no clear order*: 'Cell', 'CellularStructure', 'Cilium', 'Liposome', 'MicroOrganism' *OrganicSolidStructure: *'Cell' < 'Body structure' < 'Organism'	medical coding and classification, medical terminologies,	'MicroscopicStructure'; 'OrganicSolidStructure'	**Galen**	1.1

'Microanatomic Structure' [i.e.: 'Macromolecular Structure' < 'Cell'] < 'Tissue' < 'Organ' < 'Organ System' < 'Body Part' - 'Body Region'; *additional types: *'Body Cavity', 'Body Fluid or Substance', 'Embryologic Structure or System',	medical coding for clinical care, translational and basic research, and public information and administrative activities	'Anatomical Structure, System, or Substance'	**NCI Thesaurus**	09.07

*No clear order: *'Body organ structure', 'Body region structure', 'Body space structure', 'Body system structure', 'Body tissue structure', 'Body wall structure', 'Cell structure', 'Developmental body structure', 'Entire anatomical structure', 'Human body structure', 'Intercellular anatomical structure', 'Non-human body structure', 'Sex structure', 'Structure of bilateral paired structures', 'Structure of multiple topographic sites', 'Structure of product of conception', 'Transplant'	clinical terms	'Anatomical structure'	**SNOMED-CT**	2009_01_31

'cell' < 'portion of tissue' < 'multi-tissue structure' < 'compound organ' < 'organism subdivision' < 'whole organism'	Zebrafish anatomy and development	'anatomical structure'	**Zebrafish anatomy and development**	1.22
			
	Xenopus anatomy and development		**Xenopus anatomy and development**	1.19

'cell' < 'portion of tissue' < 'multi-tissue structure' < 'compound organ' < 'organism subdivision' < 'body'	multispecies fish anatomy	'anatomical structure'	**Teleost anatomy and development**	1.120

'Biological small molecule' < 'Biological Macromolecule' < 'Cell part' < 'Cell' < 'gross anat structure' < 'Organism'; *with 'gross anat structure' comprising: *'Organ part' < 'Organ' < 'Body part subdivision' < 'Body part' < 'Body'	vertebrate anatomy; derived mainly from FMA	'Biological object''	**Basic Vertebrate Anatomy**	1.1

*No clear order: *'anatomic region', 'body fluid or substance', 'organ system'	anatomical dictionary for the adult mouse	'adult mouse'	**Mouse adult gross anatomy**	1.195

*No clear order: *'adipose tissue phenotype', 'cardiovascular system phenotype', 'cellular phenotype', 'craniofacial phenotype', 'digestive/alimentary phenotype', *etc*.	standard terms for annotating mammalian phenotypic data	'mammalian phenotype'	**Mammalian phenotype**	1.298

*No clear order: *'anatomical system', 'embryonic structure', 'larval structure', 'tissue'	a structured controlled vocabulary of the anatomy of Amphibians	-	**Amphibian gross anatomy**	1.8

'Anatomy', 'Cell', 'Nucleus'; *with 'Anatomy' comprising: *'axis', 'body region', 'extracellular component', 'organ', 'organism'	anatomy of *C. elegans*	'C. elegans Cell and Anatomy Ontology'	***C. elegans *gross anatomy**	1.31

'cell component' < 'multi-cell-component structure' < 'cell' < 'portion of tissue' < 'multi-tissue structure' < 'organ system subdivision' < 'organ system' < 'organism subdivision' < 'organism'; *additional types: *'acellular anatomical structure', 'anatomical structure with acellular and cellular components', developing anatomical structure', 'endocrine organ', 'cell cluster organ', 'compound cell cluster organ', 'sense organ'	anatomy of *Drosophila melanogaster*	'anatomical structure'	***Drosophila *gross anatomy**	1.30

'cell component' < 'cells' < 'portion of tissue' < 'multi-tissue structure' < 'compound organ' < 'organism subdivision' < 'multi-cellular organism' *additional types: *'acellular anatomical structure', 'anatomical group', 'egg', 'extraembryonic structure',	a structured controlled vocabulary of the anatomy of mosquitoes.	'anatomical structure'	**Mosquito gross anatomy**	1.10

'acellular anatomical structure', 'portion of organism substance', 'whole organism'; *remarkably with *'organ system' *as a subtype of *'whole organism'	an ontology for spider comparative biology including anatomical parts, behavior, and products	'anatomical entity'	**Spider Ontology**	1.17

'cell component' < 'cell' < 'portion of tissue' < 'multi-tissue structure' < 'compound organ' < 'organism subdivision' < 'multi-cellular organism' *additional types: *'acellular anatomical structure', 'anatomical group', 'bar', 'carina', 'extraembryonic structure', 'inflection', 'ruga', 'sculpture', 'sense organ', 'sulcus'	anatomy of Hymenoptera	'anatomical structure'	**Hymenoptera Anatomy Ontology**	SVN Revision 2804

'cell component' < 'cell' < 'portion of tissue' < 'multi-tissue structure' < 'compound organ' < 'organism subdivision' < 'multi-cellular organism' *additional types: *'acellular anatomical structure', 'anatomical group', 'embryonic structure', extraembryonic structure',	anatomy of Bilateria	'anatomical structure'	**Bilateria anatomy**	-

'plant cell' < 'tissue' < 'organ' < 'whole plant' *additional types: *'gametophyte', 'in vitro cultured cell, tissue and organ', 'sporophyte'	botanical anatomy & morphology	'plant structure'	**Plant structure**	1.64

*No clear order: *'composite structure', 'fruitbody', 'hypha', 'mycelium', 'pseudohypha', 'sporophore', 'unicellular structure'	a structured controlled vocabulary for the anatomy of fungi.	'microbial structure ontology'	**Fungal gross anatomy**	1.3

*fiat object part: *'Regional Part Of Cell Component' < 'Regional Part Of Cell' *object: *'Molecule' < 'Cellular Component' < 'Cell' - 'Extracellular Structure' *object aggregate: *'Aggregate Object' < 'Population'	biomedical structures from macromolecules to supracellular level	'fiat object part', 'object', 'object aggregate'	**Subcellular Anatomy Ontology (SAO)**	1.2.5

'cell component' < 'cell' < 'portion of tissue' < 'multi-tissue structure' < 'compound organ' < 'organism subdivision' < 'multi-cellular organism' *additional types: *'acellular anatomical structure', 'anatomical group', 'extraembryonic structure',	cross-species anatomy: platform for integrating different species-specific anatomy ontologies	'anatomical structure'	**Common Anatomy Reference Ontology (CARO)**	1.3

*No clear order: *'acellular anatomical structure', 'anatomical group', 'anepisternum', (...), 'cell', 'cell part', 'cerebellum lobule vi', etc.	multi-species anatomy, generated from union of existing species-specific anatomy ontologies	'anatomical structure'	**Uber anatomy ontology (UBERON)**	1.72

*No clear order: *'appendage', 'body part', 'cardiovascular system', 'craniofacial tissue', 'developmental tissue - animal', 'digestive system', 'fat tissue', etc.	Minimal set of terms for anatomy	'animal component'	**Minimal anatomical terminology (MAT)**	1.1

'subatomic particle' < 'atom' < 'molecular entity' < 'poly molecular composite Entity' [i.e. 'molecular complex' < 'structured nonbiological entity'/'structured biological entity' [i.e. 'cellular component' < 'cell' < 'organism part' < 'organism']]	biomedicine	'material entity'	**BioTop**	-

Various ontologies and their partly implicit, partly explicit levels of granularity of anatomical structures (from [50]).

### 4.1 Kumar et al.'s Theory of Granularity and its Problems

One of the few formal theories of granularity with explicitly stated formal granularity relations and explicitly ranked levels of granularity that is applicable to biological objects has been proposed by Kumar et al. [13]. By applying their theory to human anatomy, the authors distinguish 12 different levels of type granularity (i.e. *Biological macromolecule *<*Subcellular organelle *<*Collection of subcellular organelles *<*Cell *<*Collection of cells *<*Tissue subdivision *<*Tissue *<*Organ part *<*Organ *<*Cardinal body part *<*Organ system *<*Organism*).

Kumar et al. [13] call objects belonging to a particular level of granularity *grains*, and all the grains of a level of granularity are marked by their own characteristic spatio-structural properties. Kumar et al. selected and demarcated different levels of granularity by paying attention to the different types of grains that exist in the biological domain of reality as well as to grain-specific basic causal principles. The reference to causal principles thereby reflects a long tradition of theories in biology about how to demarcate natural classes or natural kinds from artificial classes, ranging from Aristotelian essentialism to modern concepts of natural kinds (see, e.g., [51-59]). Kumar et al. [13] suggest seven basic principles of granularity:

*1. "Each level of granularity is determined by a class or type of *grain.

*2. The grains in a given level are parts of the grains in the next higher level*.

*3. Every level of granularity is such that summing all the grains together yields the entire human body*.

*4. The grains in a given level do not need to be all of the same size, neither do they need to be homogeneous*.

*5. The grains in a given level must be smaller in size than those entities on the next higher level of which they are parts*.

*6. With each level of granularity there is associated some specific type of causal understanding and thus some specific family of causal laws; when one moves up a level, then the grains on the lower levels become causally irrelevant*.

*7. Some entities can change through time in such a way that one and the same entity (an embryo, a tumour, an organism) can occupy a sequence of different levels of granularity in succession." *([13], p. 502).

Regarding principles 4 and 5, the authors explain that the criterion of size for drawing dividing lines between levels of granularity must be applied to each specific entity or group of specific entities in succession and not to the totality of all entities in a given level - it refers to the actual situation found in a particular specimen and its organization [13], and thus refers to instance granularity. When comparing their granularity scheme for human anatomy to their granularity principles, Kumar et al. [13] defend their scheme since only the levels of *cardinal body parts *and *organ systems *would violate principles 2 and 5, because they involve grains which overlap in the sense of sharing common parts (e.g. in humans, part of the respiratory system is in the head, another part is within the chest).

However, the applicability of Kumar et al.'s principles of granularity is highly questionable: Especially Kumar et al.'s third principle is not conform with anatomic reality. As already discussed above, in anatomy, the sum of all grains of a given level does not always yield the entire organism - independent of instance or type granularity. Just think of body cavities and microflora, which are usually not included in any level of granularity [32]. Moreover, considering the genuine cumulative nature of the anatomical organization of multicellular metazoans, this principle also fails the test of reality: usually, not all the cells of a multi-cellular organism are organized within organs. As a consequence, summing up all organs will not yield the entire body (e.g. erythrocytes, coelomocytes, and leukocytes are not part of any organ but part of a human body). In this respect, the two additional and more general principles suggested by Kumar et al. ([13], p. 504) later on in their paper, to which they also provide formal notations, seem to match better with biological reality:

1. "[F] or each instance of each universal existing at a level of granularity lower than the highest level, there is an instance of a universal at some higher (coarser) level of which the given instance is a part."

*2. "For each instance of a universal at some level of granularity higher than the lowest level, there is an instance of a universal at some lower (finer) level that is a part thereof." *([13], p. 504).

The size-principles four and five can be criticized as well [32], since using physical size only causes problems, such as the violation of principles already mentioned by Kumar et al. themselves, which, according to Keet, have to be resolved instead of handled as exceptions. Last but not least, by limiting their theory of granularity to mereological relations only, Kumar et al.'s second principle can be criticized for unnecessarily constraining the generality of their theory of granularity [32].

In addition to the seven principles, Kumar et al. also define a simple general granularity function ***gr***, where *GRAN *is the ordered set of levels of granularity (*G*_1_, *G*_2_, *G*_3_, ..., *G*_12_) applicable to a particular domain and *U *is the set of biological universals, such that ***gr ***is the function of *U *onto *GRAN*. ***gr ***returns the level of granularity where a particular entity resides:

The problem with the function is that it assumes that the domain has been partitioned already (*GRAN*) and leaves open the question of how the entities are assigned to a level - the function requires the existence of an a priori granulated domain knowledge [32,60]. Its domain-dependency limits the reusability, flexibility, and interoperability of respective computational implementations [60]. Moreover, since ***gr ***returns only one level at a time, it does not allow the organization of domain knowledge according to different perspectives of granularity within the same ontology [32], as for instance according to properties of anatomical form on the one hand, and causal dispositions (i.e. functions) or developmental properties of biological entities on the other hand.

### 4.2 Keet's General Formal Theory of Granularity

With her formal theory of granularity, Keet [32,60,61] provides an interesting alternative that seems to circumvent some of the problems associated with Kumar et al.'s [13] theory of granularity. According to Keet [32], granularity involves modeling something according to certain criteria. Each model, together with its specific criteria, forms a granular perspective. Thereby do lower levels within a perspective contain knowledge (i.e. entities, concepts, relations, constraints) or data (i.e. measurements, observations) that is more detailed than the next higher level, and higher levels simplify or make indistinguishable finer-grained details - higher levels abstract away finer-grained details [61].

The most important aspect of Keet's theory is that it allows the coexistence of different *perspectives of granularity *within a granularity framework. For example a granular perspective of relative location (i.e. based on spatial partitions) with its specific levels of granularity, along side with a perspective of structural composition (i.e. based on compositional partitions), one of biological processes (i.e. based on temporal partitions), one of causal dispositions (i.e. based on functional partitions), and a granular perspective based on developmental relations. The possibility that different granular perspectives can model a domain has been recognized before (e.g., [62]; *sorts of partitions *[14]; *multiple valid partonomic hierarchies *[63]; *perspectives *&*partitioning frame *[64]; *viewpoint dependency of hierarchy *[43]). Keet [32], however, provides the first formal general theory of granularity that incorporates different perspectives within a single *domain granularity framework*.

Keet [32] argues that the differentiation of different perspectives of granularity within a domain granularity framework implies that one can granulate a domain of reality according to different *types of granularity*. Granulating reality according to different types of granularity requires the existence of a certain type of relation that must be specific to each particular granularity perspective. This *granulation relation *relates entities/types of adjacent granular levels with one another within each perspective. Therefore, a granularity perspective is specified and can be identified by the combination of a granulation criterion and a specific type of granularity [32].

Keet [32] not only proposes a more general approach to granularity than the theory suggested by Kumar et al. [13], but also provides the respective formal definitions, axioms, and theorems accompanying her theory. Her theory is not only more general due to the fact that it allows the combination of different perspectives of granularity within a single framework, but also because it is not solely based on parthood relations (i.e. mereology). It considers taxonomic inclusion (i.e. class-subsumption hierarchy based on is-a relations and thus on set theory) as another distinct but valid way of understanding granularity (see also [61]). Moreover, it accommodates both quantitative (i.e. arbitrary scale) and qualitative (i.e. non-scale dependent) aspects of granularity.

According to Keet, a granularity framework must meet the following criteria:

• A granularity perspective must have at least two levels of granularity.

• The entities/types in a granular level must have at least one aspect in common, which represents the *criterion of granulation *by which to granulate the data, information, or knowledge [32].

• The criterion of granulation specifies the kind/category of properties according to which the domain is partitioned, levels identified, and the subject domain is granulated.

• A particular granularity level may only be contained in one perspective [65].

• Given that each granular level is contained in a granular perspective and different perspectives within a domain granularity framework are disjoint, a particular entity/type may reside in more than one level, but all the levels in which it is contained must belong to distinct granular perspectives.

The advantage of Keet's approach is obvious. The increase in generality coupled with the increase in formalism results in more flexibility and thus a broader applicability of her theory within knowledge management systems that use ontologies. Keet's general theory of granularity allows for a detailed and sophisticated structuring of an ontology by separating types of entities into various different types of taxonomic and partonomic hierarchies [32]. Like a meta-ontology, this additional organizational layer can be formally added onto an existing ontology. The precise categorization of ontology components by type according to, for instance, relative location, biological processes, structural components, causal dispositions (i.e. functions), structural and functional parthood relations, developmental properties and relations, genealogical relations, and evolutionary origin, facilitates the construction of a more realistic and detailed model of biological reality.

## 5 General Schemes for Partitioning Complex Structures

In the following I will distinguish three different approaches of decomposing a complex structure solely based on its ***spatio-structural properties***, each of which results in a specific type of perspective. These three basic types of decompositions - compositional, spatial, and resolution-dependent - serve as a basis for distinguishing three corresponding general types of type granularity perspectives, which will be discussed in a subsequent section. Thereby, for the purpose of this paper, the **Basic Formal Ontology **(BFO [3]) will be used as a top-level ontology. Reference will be made to its defined type 'material entity' (i.e. a spatially extended material entity) and its subtypes 'object' (i.e. a material entity that is maximally self-connected, possessing an internal unity), 'fiat object part' (i.e. a material entity that is part of an object but not demarcated by any physical discontinuity), and 'object aggregate' (i.e. a material entity that is a sum of separate object entities, possessing non-connected boundaries) (see Table 2 for complete definitions).

**Table 2:** Definitions of the Basic Formal Ontology.

Definition	Parent Class Affiliation	Link/ID
**'material entity': ***"An independent continuant that is spatially extended whose identity is independent of that of other entities and can be maintained through time. Note: Material entity subsumes object, fiat object part, and object aggregate, which assume a three level theory of granularity, which is inadequate for some domains, such as biology*. *Examples: collection of random bacteria, a chair, dorsal surface of the body"*	'independent continuant'	http://www.ifomis.org/bfo/1.1/snap#MaterialEntity

**'object': ***"A material entity that is spatially extended, maximally self-connected and self-contained (the parts of a substance are not separated from each other by spatial gaps) and possesses an internal unity. The identity of substantial object entities is independent of that of other entities and can be maintained through time*. *Examples: an organism, a heart, a chair, a lung, an apple"*	'material entity'	http://www.ifomis.org/bfo/1.1/snap#Object

**'fiat object part': ***"A material entity that is part of an object but is not demarcated by any physical discontinuities*. *Examples: upper and lower lobes of the left lung, the dorsal and ventral surfaces of the body, the east side of Saarbruecken, the lower right portion of a human torso"*	'material entity'	http://www.ifomis.org/bfo/1.1/snap#FiatObjectPart

**'object aggregate': ***"A material entity that is a mereological sum of separate object entities and possesses non-connected boundaries*. *Examples: a heap of stones, a group of commuters on the subway, a collection of random bacteria, a flock of geese, the patients in a hospital"*	'material entity'	http://www.ifomis.org/bfo/1.1/snap#ObjectAggregate

Definitions of the top-level types of material entity of the Basic Formal Ontology (BFO version 1.1).

### 5.1 Compositional versus Spatial Partitions

**Figure 3:**
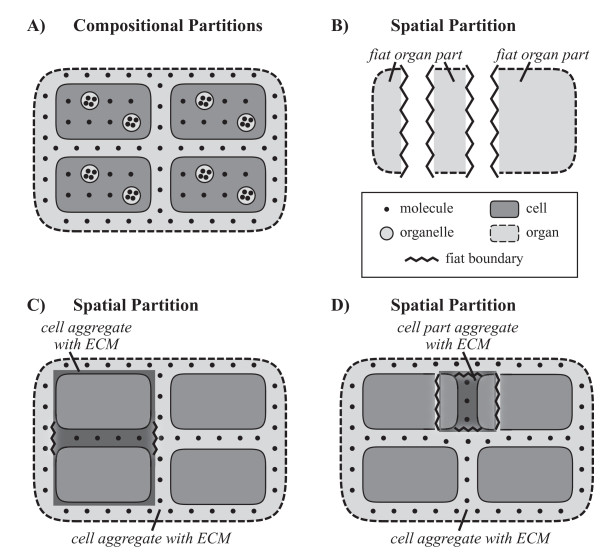
**Examples of Compositional and Spatial Partitions**. **A) **A combination of *compositional partitions *of an object into objects of a lower level of granularity as its *constitutional parts*. Exemplified by three partitions of an organ: A partition into cells, one into organelles, and one into molecules. **B) **A *spatial partition *of an object into fiat object parts as its *regional parts*. Exemplified by the partition of an organ into three fiat organ parts. **C) **A *spatial partition *of an object into fiat object parts as its *regional parts*. Exemplified by the partition of an organ into two cell aggregates with ECM. **D) **A *spatial partition *of an object into fiat object parts as its *regional parts*. Exemplified by the partition of an organ into a cell part aggregate with ECM and a cell aggregate with ECM. **ECM**: extracellular matrix.

While discussing different ways of partitioning anatomical structures, Mejino et al. [5] distinguish a compositional from a spatial partition (see also [11,66]). The compositional partition is based on what they call *constitutional parts*, which are distinct (anatomical) objects that are components of a structural (anatomical) whole, and thus of an object of a higher granularity level (Fig. 3A). A compositional partition thus describes the component parts of a given object, which themselves are objects of a lower level of granularity. In other words, whenever a given object is partitioned and the resulting set of parts are exclusively objects of a lower level of granularity, it represents a compositional partition. This also holds for the partitioning of object aggregates into their constitutional object parts.

Mejino et al.'s [5] spatial partition (see also *fiat partition*, [66]), on the other hand, is based on *regional parts*. Regional parts are fiat object parts that result from an arbitrary subdivision of an (anatomical) object into sets of diverse constitutional parts that share a given location within and relative to the object (Fig. 3B, C, and 3D). A spatial partition thus describes the fiat object parts that result from an arbitrary spatial division of an object. Although arbitrary, the resulting fiat boundaries nevertheless may be determined by specific landmarks or coordinates [67], or other pragmatic or even scientifically justified reasons, like for example differences in genetic expression patterns of cells of different organ subdivisions. Anyhow, whenever a given object is partitioned and the resulting set of parts consists of fiat object parts, it represents a spatial partition. The demarcation of compositional and spatial partitions seems to be straight forward: If a partition includes only fiat object parts, it represents a spatial partition, and if it includes only objects, it represents a compositional partition. However, do these two cases cover all possible spatio-structural partitions?

#### 5.1.1 The Special Case of 'Object Aggregate' in Cumulative Constitutive Hierarchies

The BFO not only distinguishes 'object' and 'fiat object part', but also 'object aggregate'. Where do object aggregates fit into this scheme? Whereas one could argue that for constitutive hierarchies, object aggregates may be treated like constitutional compositions (i.e. with objects as proper parts of object aggregates or object aggregates as proper parts of objects that belong to the adjacent higher granularity level), for cumulative constitutive hierarchies this is more problematic. This is because aggregates of objects of cumulative-constitutively organized entities are composed not only of the objects defining the aggregate, but also of objects and fiat object parts of lower levels of granularity. A cell aggregate of a multicellular metazoan, for instance, is not only composed of a set of cells, but also of the molecules that form the extracellular matrix (ECM). The ECM surrounds almost all cells of multicellular metazoans, but is not part of cells itself. Instead, it forms a continuous matrix, and the demarcation of cell aggregates within this matrix is always accompanied by fiat boundaries through the ECM (Fig. 3C). As a consequence, an aggregate of (bona fide) cells and a fiat portion of ECM is not covered by (i.e. does not instantiate) BFO's concept of 'object aggregate', because the former includes fiat object parts and the latter does not. Instead, it is instantiated by BFO's concept of 'fiat object part'. Therefore, any partition of an entity into such fiat aggregates is a spatial partition.

From this follows, unfortunately, that for cumulative-constitutively organized material entities the concept of 'object aggregate' is of limited applicability. In biology, its applicability is almost exclusively restricted to the molecular level. Although biologists usually talk about some anatomical structures as if they are object aggregates (e.g. *cell cluster*, *cell aggregate*), the respective structures nevertheless are fiat object parts, because they possess fiat boundaries. Ontologies should therefore better refer to such fiat aggregates as 'object aggregate with fiat object part', as for example 'cell aggregate with ECM', instead of 'cell aggregate' for a cluster of cells embedded in ECM (Fig. 3C), and they must subsume them under 'fiat object part'.

#### 5.1.2 Criteria for Compositional Partitions

A compositional partition can be defined by referring to a proper parthood relation with the restriction that this relation exists only between objects that are proper parts of other entities (i.e. objects, object aggregates, or fiat object parts). Moreover, the constitutive parts of a given compositional partition may not be proper parts of one another (i.e. they must be *direct proper parts*). For the case of an organ of a multicellular metazoan, one can thus distinguish a cellular, an organelle, and a molecular compositional partition. Thereby, due to the organ's cumulative constitutive organization, only the molecular partition is exhaustive - the collection of all cells of a multicellular metazoan, for instance, does not sum up to the entire organism (the ECM, e.g., is not included in this collection but nevertheless belongs to the organism).

Since any partition with objects as its parts represents a compositional partition, depending on what is partitioned into its object components, one can distinguish three basic types of compositional partitions: (i) a *compositional object partition*, in which objects are partitioned into their constitutive object parts; (ii) a *compositional object aggregate partition *(with limited applicability for biological and all other cumulative-constitutively organized material entities), in which object aggregates are partitioned into their constitutive object parts; and (iii) a *compositional fiat object part partition*, in which fiat object parts are partitioned into their constitutive object parts, while ignoring the left over fiat object part components.

Of these three basic types, the compositional object partition takes in a special position: Since compositional parts are objects themselves, they can also be partitioned into their constitutive parts. This results in a hierarchical system of compositional parthood relations, in which the constitutive parts of one level become the objects of partition of the next lower level. Such combinations of different compositional partitions can be organized as an encaptic hierarchy of proper parthood relations - a granular partition. This granular partition can be represented as a tree of proper parthood relations between objects, with each compositional object partition contributing one level of constitutive parts, thereby forming what Reitsma and Bittner [36] call a *bona fide granularity tree*. Regarding Keet's theory, on the other hand, the combination of compositional object partitions form a compositional object granularity perspective.

#### 5.1.3 Criteria for Spatial Partitions

The situation is different when partitioning a complex whole into its regional parts. For defining a particular spatial partition it is not sufficient to refer to a proper parthood relation that has a fiat object part assigned to it, because partitioning a given material entity into all of its possible fiat object parts will yield many different but ontologically still equally well justified spatial partitions. Unfortunately, due to the arbitrariness of fiat boundaries, these partitions are usually not congruent with one another - *"Reality is a cheese: it can be cut in many ways." *([68], p. 21). As a consequence, for a given object there are always more spatial partitions possible than compositional partitions. Moreover, each spatial partition forms a granularity level and not all possible levels can be combined to a single spatial granularity perspective. Put differently, there always exist several spatial granularity perspectives that cannot be mapped onto one another. Therefore, due to the arbitrariness of fiat boundaries, spatial granularity perspectives always require criteria additional to being spatial partitions.

One possibility is to link a spatial partition to a given compositional partition. A spatial partition could thus be defined in reference to a proper parthood relation that is restricted to hold only between a fiat object part and its corresponding object. However, this parthood relation requires additional constraints: the regional parts of such a spatial partition must represent proper parts of objects belonging to the same compositional partition (i.e. objects of the same compositional granularity level). While this would still result in many different partitions, one could differentiate between the different granularity levels of the objects to which they refer - to each object granularity level a set of possible spatial partitions would be assigned.

#### 5.1.4 Combining Compositional and Spatial Partitions

The **Foundational Model of Anatomy **ontology (FMA [11,62]) represents a well developed and frequently used ontology for human anatomy. It has been used as a template for developing vertebrate anatomy ontologies as well. Implicitly, it uses a type granularity framework that combines a compositional and a spatial granularity perspective in which the spatial partition is dependent on the compositional partition. The backbone hierarchy of the FMA is provided by what the authors [11] call *salient levels of structural organization *(*Biological macromolecule *<*Cell *<*Portion of tissue *<*Organ *<*Organ system *<*Cardinal body part *<*Body*). According to Rosse and Mejino they are defined in reference to *units of structural organization *that possess only bona fide boundaries (i.e. objects and object aggregates). Since the levels are conceived to contain only objects and object aggregates, the respective partition would represent a compositional partition. In addition to this compositional partition, the FMA distinguishes what the authors call *transitional *or *intermediate levels *(*Cardinal cell part *<*Cardinal tissue part *<*Cardinal organ part *<*Organ system subdivision *<*Subdivision of cardinal body part*). They are defined in reference to *subdivisions *or *cardinal parts *of objects and thus in reference to the *salient levels of structural organization*. Since these transitional levels are conceived to contain only fiat object parts, the respective partition would represent a spatial partition. And since they are defined in reference to the salient levels, each spatial partition is linked to a corresponding compositional partition, which at its turn provides the necessary additional criterion.

Interestingly, Rosse and Mejino [62] interpret the transitional levels to represent intermediates that provide the connection between the salient levels. Thus they seem to implicitly combine these two granularity perspectives within a single granularity framework. According to Keet's general formal theory of granularity [32], such a combination of different granularity perspectives within the same granularity framework is possible, as long as the perspectives are clearly separated and no granularity level is part of more than one granularity perspective.

The granularity scheme of the FMA also bears some problems that result from the cumulative constitutive organization of multicellular organisms and the problems of would-be "object aggregates" within such organizations. Most of the time they actually represent fiat object parts and therewith regional parts rather than constitutional parts, as they are *object aggregates with fiat object parts*. For instance are FMA's 'Portions of tissue', 'Organ system', and 'Cardinal body part' not instantiated by objects or object aggregates but actually by fiat object parts, which do not represent constitutional parts. Instead, they represent regional parts and can therefore only belong to spatial and not to compositional partitions. As a consequence, FMA's salient levels of organization contain levels defined in reference to constitutive parts alongside with levels defined in reference to regional parts. Such a mixture of compositional and spatial partitions within one granularity perspective, however, is formally inconsistent and should be avoided.

Since FMA's transitional levels are defined in reference to their respective salient levels, this problem also affects the transitional levels: 'Cardinal tissue part', 'Organ system subdivision', and 'Subdivision of cardinal body part' are instantiated by regional parts of fiat object parts and therewith by regional parts of regional parts. As such, they are lacking the necessary additional criterion, since they are not defined in reference to a respective granularity level of a compositional perspective.

A similar problem applies to Kumar et al.'s [13] granularity scheme for human anatomy. It is responsible for their levels of 'cardinal body part' and 'organ system' failing to meet their granularity principles two (i.e. *grains in a given level are parts of grains in the next higher level*) and five (i.e. *grains in a given level must be smaller in size than those entities on the next higher level of which they are parts*). Part of the human respiratory system is located in the head and another part is within the chest, because cardinal body parts and organ systems obviously belong to two distinct and incongruent regional partitions and thus to two different spatial granularity perspectives. This becomes also apparent when considering the granular partitions criterion of non-partial overlap mentioned earlier (see *2.1 Granular Partition and Granularity Tree*), since it implies that the partition of human anatomy into organ systems and into cardinal body parts together do not form a granular partition and thus also no granularity tree. One has to keep in mind that levels belonging to different perspectives should never be combined within a single *synthetic *perspective but rather within a general granularity framework.

#### 5.2 Scale-Based versus Qualitative Partitions

A notion commonly associated with granularity is that of size. For example, it is often claimed that entities belonging to the same level of granularity should have roughly the same size. While such requirements have been identified to be problematic (see, e.g., Kumar et al.'s fourth principle: *grains in a given level do not need to be all of the same size *[13]), size relations still seem to be important to theories of granularity (Kumar et al.'s fifth principle: *grains in a given level must be smaller in size than those entities on the next higher level of which they are parts *[13]). However, one has to be very careful when requiring specific size relations in connection with granularity schemes. The actual size values may vary significantly not only among the particular instances of a given type but also among different moments in time for the same individual entity. Therefore, size relations should always refer to actual conditions and the organization found within one and the same particular entity at a specific moment in time and should not be claimed to exist time-independently and between parts belonging to different particular entities [13].

Rector et al. [69] distinguish *qualitative *from *quantitative *granularity and recommend to use the term *collectivity *for qualitative granularity and the term *size range *for scale-based granularity. According to Rector et al. [69], a *collective *represents an emergent whole which consists of *grains *that represent the granular parts of the collective. In other words, entities considered individually at one level of granularity are considered as collectives with emergent properties at the next higher level, and between entities of lower and higher levels exist part-whole relations. The two types of granularity perspectives discussed above, based on regional and constitutive parthood relations, represent examples for qualitative granularity. According to Rector et al. [69], scale-based *size range*, on the other hand, is not based on a parthood relation between grains and collectives but on a relation of *large *and *small*: grains cannot be physically larger than their collectives.

But then, Keet [32] distinguishes between *scale dependent *and *non-scale dependent *granularity perspectives, which she treats as fundamentally different types of granularity perspectives within her formal theory of granularity. As she notes, *"[f] or scale-dependency it is important to recognise that it is the *representation *of the entity (/type) that counts. The representation of the real-world entity (/type) is different at different levels of granularity and the representation is the resultant of the *combination *of the entity (/type) and the scale at which it is considered (...)" *(emphasis taken from the original; [32], p. 84). According to her taxonomy of types of granularity, Keet [32,65] distinguishes between *grainsize *(i.e. scale on entity), with the subtypes *resolution *(e.g. cell wall as line, lipid bi-layer, or 3D structure) and *size of the entity *(e.g. coin separator of a vending machine) on the one hand, and *aggregation *(i.e. scale of entity), with the subtypes *overlay aggregated *(e.g. map of earth with more/less isotherms) and *entities aggregated according to scale *(e.g. second, minute, hour) on the other hand, as two basic types of scale dependent granularity. The criterion of granulation of scale dependent granularity perspectives is a combination of a quality property and a sortal property, as for instance the surface of a structure and a surface metric scale. The relation between entities belonging to adjacent levels of scale dependent granularity thereby maps onto a parthood relation [32] that combines proper parthood with a math function for conversion of units of measurement.

#### 5.2.1 Why We Need Scale Dependent Granularity

The importance of scale dependent granularity perspectives results from pragmatic considerations regarding the overwhelming richness in detail that is inherent to nature and the fundamental cognitive and general epistemological constraints that every particular scientist and every actual scientific enterprise has to deal with. For example, if one wants to investigate the social behavior of a specific individual animal, as a first step it would usually not be very efficient to analyze its total structural composition on a molecular level. This would not only consume too much time and money (two very valuable resources), but would produce also significant amounts of irrelevant data. Thus, depending on the purpose of an investigation, one usually ignores specific levels of detail (i.e. levels of granularity) and focuses on others. If a morphologist studies the anatomy of a multicellular metazoan specimen using gross anatomy preparation methods, she usually cannot make any statements about the number and spatial arrangements of individual cells. At this (granular) level of resolution, she can only speak of cell aggregates as certain *portions of tissue*, which represent certain *amounts *or *portions of matter*.

**Figure 4:**
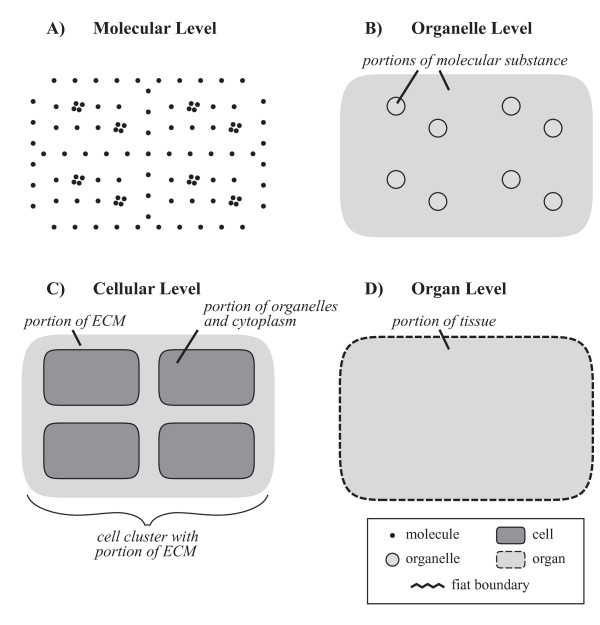
**Scale dependent Resolution**. **A) **On the *molecular level*, individual molecules and aggregates thereof can be distinguished and are referred to by using count-nouns (i.e. 'molecule'; 'molecule aggregate'). **B) **On the *organelle level*, individual organelles can be distinguished and are referred to by using count-nouns (i.e. 'organelle'). Individual molecules and aggregates thereof, however, are usually not distinguished anymore and one refers to them by using mass-nouns, such as 'portion of molecular substance'. One can distinguish extra-organelle from intra-organelle portions of molecular substance. **C) **On the *cellular level*, individual cells can be distinguished and are referred to by the count-nouns 'cell' or 'cell cluster with ECM' (or its resolution-dependent countable representation 'cell cluster with portion of ECM'). Individual organelles, however, are usually not distinguished anymore. Instead, one refers to the extracellular matter as non-countable 'portion of ECM', whereas the intracellular matter represents a granular mixture of non-countable organelles and cytoplasm and is referred to as 'portion of organelles and cytoplasm'. Both represent mass-nouns. **D) **On the *organ level*, individual cells are usually not distinguished anymore. Instead, one refers to the matter contained in organs as a 'portion of tissue', which is the resolution-dependent non-countable representation of 'cell aggregate with ECM'. 'Portion of tissue' therewith represents a mass-noun. **ECM**: extracellular matrix.

Portions of matter do not represent countable units (i.e. count-nouns), but require some arbitrary, although standardizable, quantification measure, like *a glass *of water or *1 m^3 ^*of soil, therewith representing what is generally referred to as *mass-nouns*. The distinction of mass-noun and count-noun, however, is not absolute, but a result of granular focus (resolution). A glass of water, for instance, which is *represented *by a mass-noun, can be decomposed on the molecular level into an aggregate of a specific number of water molecules, which at its turn is a *representation *that uses a count-noun. Whether a count-noun or a mass-noun is used depends largely on the individual interests and purposes of the investigator: if one is interested in the molecular composition of a particular wine, one would treat it as a discrete aggregate of molecules, whereas if one is only interested in consuming a certain amount of it, one treats it as a continuous aggregate that has to be partitioned according to volume measures. The same applies when investigating the anatomy of multicellular metazoans: what represents a *portion of tissue *at a coarser level of resolution/granularity can be decomposed on a finer grained level into a *cell aggregate with a portion of molecular substance*: a specific number of individual cells accompanied by a portion of ECM. Therefore, the distinction between *portion of tissue *and *cell aggregate with portion of ECM *is not a matter of qualitative properties but only a matter of resolution and, thus, of scale (Fig. 4).

Obviously, contrary to non-scale dependent granularity, scale dependent granularity is not always exclusively based on properties of material entities that exist independently of human activities. Some types of scale dependent granularity, as for example resolution granularity, are based on properties and relations resulting from the interaction of a human being with the material objects around her (including other human beings and herself). This involves epistemological and cognitive constraints that affect our *representations *of real entities. Whereas this might tell us more about our cognitive system than about the entities themselves, it is nevertheless real and we have to deal with it when doing science. Therefore, it is advisable to carefully distinguish such *epistemological *types of scale dependent granularity, which depend on peculiarities of human cognition (e.g., *resolution*), from *ontological *types of scale dependent granularity (e.g., *size of the entity*). Anyhow, one must realize that although quantitative scale dependent and qualitative non-scale dependent types of granularity represent effectively independent types of granularity perspectives [69], and epistemological and ontological types of scale dependent granularity must be distinguished carefully. All of them are essential and required in scientific research practice.

#### 5.2.2 'Object Cluster' versus 'Portion of Substance'

Considering resolution and the resulting granularity relation between count-nouns and mass-nouns, and considering the specific conditions found in cumulative constitutive organizations, it follows that all entities that are object aggregates or that are fiat object parts that possess aggregates of objects as their parts (e.g. cell aggregates with ECM) can be represented in two different ways.

The example from above with a countable and a non-countable representation of *cell aggregate with ECM *(Fig. 4) shows: While in reality the entities of a particular type of 'cell aggregate with ECM' that are associated with different resolution levels are the same, their ***representation ***changes according to resolution. A cell aggregate with ECM can be represented with two different types of resolution-dependent concepts - two mappings from the same real world entity. Put differently, it instantiates two different types of representation (see [32]) - a type for which the amount of cells in the aggregate is countable and a type for which it is non-countable.

This holds not only for fiat object parts that possess object aggregates as their parts, but also for object aggregates. Both require at least two distinct representations. In case the objects in the aggregate are non-countable, the term 'portion of substance' should be used for the coarser grained representation. In case the objects in the aggregate are countable and one refers to them as a countable cluster of objects, the term 'object cluster' should be used for object aggregates and 'object cluster with portion of substance' for object aggregates with fiat object parts for the finer grained representation. Thus, for instance an aggregate of six cells embedded in ECM should be referred to as a 'cell cluster with portion of ECM' if one refers to the cells as individual objects. However, if the presence of the individual cells is irrelevant, one should use the term 'portion of tissue', which represents the coarser grained representation. Anyhow, since both 'cell cluster with portion of ECM' and 'portion of tissue' are alternative types of representation of a cell aggregate with ECM, one could also refer to the latter if one does not want to account for resolution. This applies to object aggregates accordingly.

## 6 A General Integrated Spatio-Structural Granularity Framework for Cumulative-Constitutively Organized Material Entities

Cumulative-constitutively organized material entities usually possess a complex structure. Modeling their structural properties more or less comprehensively requires a general theory of granularity that allows to combine several different type granularity perspectives within one integrated type granularity framework. In order to develop such an integrated type granularity framework one first has to identify all relevant basic types of material entity and then analyze all possible qualitative and scale dependent relations among them that are relevant for modeling their spatial organization - their form or morphology. In other words, all relevant major compositional and spatial partitions have to be identified and formally covered, and where appropriate, modeled as separate type granularity perspectives.

### 6.1 Why Not Kumar et al.'s Theory of Granularity

The granularity theory of Kumar et al. [13] does not meet the criteria required for such a task. Their second principle of granularity (i.e. *grains in a given level are parts of the grains in the next higher level*) constrains granularity on qualitative parthood relations (i.e. mereology-only relations) and thus excludes the possibility to model scale dependent granularity such as resolution. Resolution, however, is a key concept in most empirical experimental sciences and should be accounted for by any model of reality. Kumar et al.'s third principle (i.e. *every level of granularity is such that summing all the grains together yields the entire human body*), although a common principle in mereological theories, would prohibit for instance a granularity level 'cell' for human anatomy, since human bodies represent cumulative-constitutively organized material entities. The sum of all cells does not yield the corresponding whole human body. This is due to the fact that, besides various types of extracellular fluids and different kinds of body cavities, a very important part of the human body consists of extracellular matrix (ECM), which holds the cells together. ECM, extracellular fluids, and body cavities, however, are not covered by an exclusively cellular model of human anatomy. Despite this inconsistency, Kumar et al. [13] themselves suggest a 'cell' granularity level and do not even discuss the resulting problems regarding their third principle. This can be understood as an indication to their third principle not being necessary. It unnecessarily restricts their granularity theory (this criticism also applies to the theory of granular partitions and granularity trees when applied to types and not to instances; see Chapter 2.1 and discussion in Chapter 7).

Since Kumar et al.'s theory of granularity is narrow in scope, constrained on parthood relations only, and, moreover, restricted to accommodate only a single granularity perspective, it is not adequate for modeling spatio-structural granularity of cumulative-constitutively organized material entities. The general theory of granularity suggested by Keet [32], on the other hand, is less restrictive and can even accommodate multiple granularity perspectives, integrated into a common granularity framework. Her theory thus provides a theoretical framework that perfectly suits the requirements of modeling spatio-structural granularity of cumulative-constitutively organized material entities.

### 6.2 Identifying and Demarcating the Different Types of Structural Granularity Perspectives

#### 6.2.1 The Subject Domain

The here proposed general integrated structural granularity framework is based on Keet's general theory of granularity [32]. Its *subject domain *is restricted to material entities that exhibit a cumulative constitutive organization. Taking the **Basic Formal Ontology **(BFO [3]) as a top-level ontology, its basic types of 'material entity' - 'object aggregate' (although of limited applicability), 'object', and 'fiat object part' - represent foundational types within the here proposed granularity framework. With them, all possible types of material entity that possess structural integrity (i.e. no part separated from all other parts by a gap) can be classified. Every possible arbitrary fiat part as well as every possible bona fide part of a cumulative-constitutively organized material entity is covered in principle.

#### 6.2.2 The Backbone: A Compositional Object Granularity Perspective

The three types of partitions discussed in the previous section (*5 General Schemes for Partitioning Complex Structures*) - two qualitative types of partitions (i.e., compositional and spatial) and one quantitative type (i.e. resolution), provide the core for the different types of granularity perspectives presented here.

**Figure 5:**
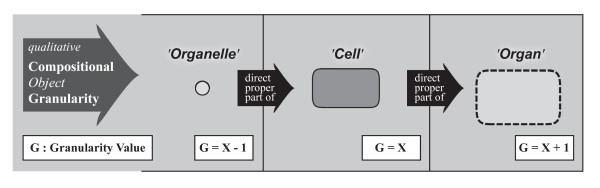
**Compositional Object Granularity Perspective**. A particular qualitative *compositional object granularity perspective *that is defined in reference to a combination of direct proper parthood and the defining properties of 'object' as its granulation criterion. The levels of granularity are demarcated according to the defining properties of different subtypes of 'object' that possess proper parthood relations to other such subtypes (e.g. *'Organelle', 'Cell', 'Organ'*), and they are ordered according to the direction of direct-proper-part-of relations.

The most important granularity perspective of the framework is based on compositional partition. It is important because it provides a reference backbone to the other perspectives (here, and in the following, if not stated otherwise, *granularity *refers to *type granularity*). This ***compositional object granularity perspective ***(short: *CO perspective*) is based on a direct proper parthood relation between different subtypes of 'object' (see Fig. 5). Thus, its *granulation criterion *combines the defining properties of 'object' with the direct proper (constitutive) parthood property:

'object'   *is-direct-proper-part-of*   'object';

'object'   *has-direct-proper-part*   'object'.

As a consequence, all objects that possess a direct proper parthood relation to at least one other object belong to this perspective.

Following Keet, the CO perspective thus has granulation of the non-scale dependent single-relation-type (*nrG *[32]) *granularity type*, also called the non-scale dependent primitive (*npG *[61]) granularity type. It uses the direct proper parthood relation as its *granulation relation*.

The different granularity levels of a CO perspective are defined and demarcated from one another in reference to the defining properties of those subtypes of 'object' of a given ontology that possess proper parthood relations to other subtypes of 'object'. As a consequence, each granularity level has at least one particular subtype of 'object' assigned to it. However, in order to constitute a granularity perspective, there have to be at least two levels of granularity and thus two distinct and disjoint subtypes of 'object' in a given ontology [32].

The lowest level of granularity refers to the particular 'object' subtype of which at least one instance is a direct proper part of an instance of another 'object' subtype and of which no instance has an instance of another subtype of 'object' as its proper part. This way, entities residing in adjacent granularity levels are related to one another according to the direct proper parthood *granulation relation*. In other words, the granularity levels are demarcated from one another according to the properties of the subtypes of 'object' of a given ontology that possess proper parthood relations and they are ordered according to the direct proper parthood granulation relation.

For *instances *of cumulative-constitutively organized material entities holds:

1. An object entity is not necessarily a proper part of some object entity that belongs to the adjacent higher level of CO granularity.

2. Every object entity that does not belong to the lowest level of CO granularity has at least two object entities as its proper parts.

3. The object entity that is granulated represents the maximum object entity belonging to the highest CO granularity level. Every other object entity belonging to this granulation is a proper part of this maximum object entity. Thus, put differently, all object entities that belong to a granulation are proper parts of one maximum object entity. However, due to the cumulative constitutive organization, the object entities that are direct proper parts of the maximum object entity not necessarily belong to the second highest level of CO granularity but may belong to any level of the granulation.

#### 6.2.3 Five Additional Compositional Granularity Perspectives

**Figure 6:**
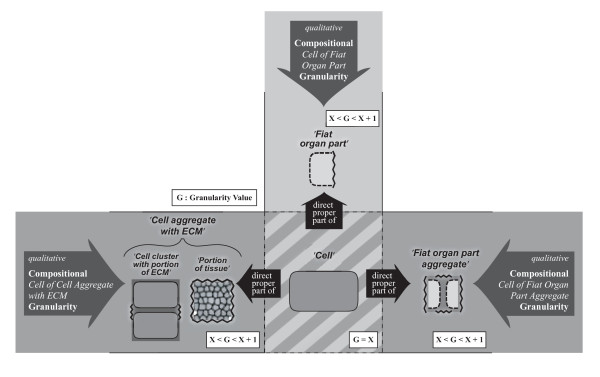
**Additional Compositional Granularity Perspectives**. A combination of three particular qualitative *compositional object of fiat object part granularity perspectives*. Each perspective is defined in reference to a combination of direct proper parthood and either the defining properties of specific subtypes of 'object' (in the perspectives depicted it is 'cell'), all of which belong to the same compositional object granularity level, or the defining properties of their respective type of 'fiat object part', of which the object entities are direct proper part of (in the perspectives depicted these are 'cell aggregate with ECM', 'fiat organ part', and 'fiat organ part aggregate'). Each of these additional compositional granularity perspectives contains only two levels: a lower level, to which subtypes of 'object' belong (e.g. 'cell'), and a higher level, to which subtypes of 'fiat object part' belong (e.g., 'cell aggregate with ECM', 'fiat organ part', or 'fiat organ part aggregate'). Notice that the three perspectives shown overlap with one another at their 'cell' levels - a particular cell can belong to all three perspectives at the same time. **ECM**: extracellular matrix.

Additionally to the compositional object granularity perspective one can differentiate five other types of compositional granularity perspectives. A ***compositional object of fiat object part granularity perspective ***that is based on a direct proper parthood relation between a specific subtype of 'fiat object part' and its corresponding parts that represent the matching subtypes of 'object' (for examples see Fig. 6). A ***compositional object aggregate of fiat object part granularity perspective***, in which the corresponding parts are the matching subtypes of 'object aggregate' based on a proper parthood relation. A ***compositional object of object aggregate granularity perspective***, which is based on a direct proper parthood relation between a specific subtype of 'object aggregate' and its corresponding subtype of 'object' as its part. And a ***compositional object aggregate of object granularity perspective***, which is based on a proper parthood relation between a specific subtype of 'object' and its corresponding subtype of 'object aggregate' as its part. Furthermore, a ***compositional object aggregate of object aggregate granularity perspective***, which is based on a proper parthood relation between specific subtypes of 'object aggregate'. In case of cumulative-constitutively organized entities, however, due to the limited applicability of the type 'object aggregate', all additional compositional granularity perspectives are of limited relevance, except for the *compositional object of fiat object part granularity perspective*.

The *granulation criterion *of each additional compositional perspective combines the (direct) proper parthood property with the defining properties of either a specific subtype of 'object' that is assigned to a particular granularity level of the CO perspective or of the specific corresponding subtype of 'object aggregate' or 'fiat object part' respectively:

i)   'object'   *is-direct-proper-part-of*   'fiat object part';

      'fiat object part'   *has-direct-proper-part*   'object'.

And of limited relevance:

ii)   'object aggregate'   *is-proper-part-of*   'fiat object part';

      'fiat object part'   *has -proper-part*   'object aggregate';

iii)   'object'   *is-direct-proper-part-of*   'object aggregate';

      'object aggregate'   *has-direct-proper-part*   'object';

iv)   'object aggregate'   *is -proper-part-of*   'object';

      'object'   *has -proper-part*   'object aggregate';

v)   'object aggregate'   *is -proper-part-of*   'object aggregate';

      'object aggregate'   *has -proper-part*   'object aggregate'.

As a consequence, only objects that belong to the same CO granularity level and their corresponding object aggregate and fiat object part counterparts belong to the same particular compositional perspective. From this follows that for object aggregates and fiat object parts there exist as many different particular additional compositional perspectives as there are different levels of CO granularity (except for the maximum object and thus the highest level of CO granularity, as its instances by definition and de facto cannot be part of any higher level object). The additional compositional perspectives are demarcated from one another according to the defining properties of the respective 'object' or 'object aggregate' subtypes that must belong to the same CO granularity level. In other words, each particular compositional perspective is either directly or indirectly assigned to a particular CO granularity level.

Each of these particular compositional perspectives has granulation of the non-scale dependent single-relation-type *granularity type *(*nrG *[32]). They use the (direct) proper parthood relation as their *granulation relation *and are in this respect compatible to the CO perspective.

Contrary to the CO perspective, however, each of these additional compositional perspectives consists of only two granularity levels (except for the compositional object aggregate of object aggregate perspective, which can have more than two levels). The granularity level to which the parts belong represents the lower level, and entities belonging to this lower level are related to entities belonging to the higher level through (direct-) proper-part-of relations.

**Figure 7:**
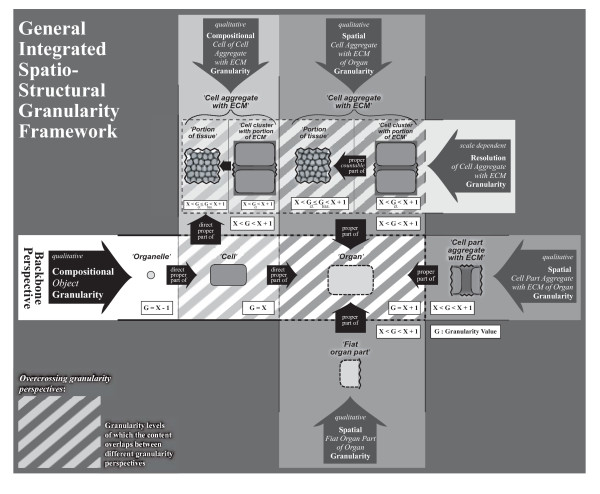
**The General Integrated Spatio-Structural Granularity Framework**. Some of its granularity perspectives are shown and how they relate, i.e. *overcross*, with one another.

**Figure 8:**
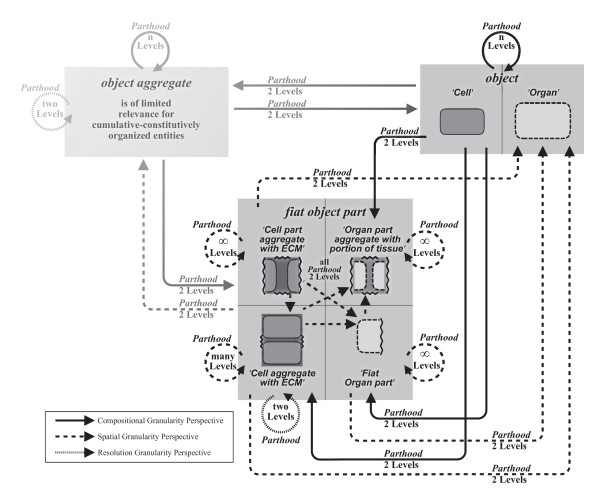
**Overview of the Framework**. Overview of the different types of compositional, spatial, and resolution granularity perspectives of the General Integrated Spatio-Structural Granularity Framework and how they relate to the basic subtypes of 'material entity', with 'Cell', 'Organ', 'Cell aggregate with ECM', 'Cell part aggregate with ECM', 'Organ part aggregate with portion of tissue', and 'Fiat organ part' as examples. The arrows indicate whether it is a compositional, a spatial, or a resolution perspective and how many granularity levels it distinguishes. Different subtypes of 'fiat object part' are distinguished (i.e. 'Cell part aggregate with ECM', 'Organ part aggregate with portion of tissue', 'Cell aggregate with ECM', and 'Fiat organ part'), which allow the differentiation of further subtypes of spatial and compositional granularity perspectives, which, however, are not discussed in detail here. All perspectives involving object aggregates are not covered in detail, since they are of limited relevance for cumulative-constitutively organized entities. **ECM**: extracellular matrix.

By distinguishing different subtypes of 'fiat object part' one can differentiate further subtypes of compositional granularity perspectives, which, however, would go beyond the scope of this paper (for examples see Fig. 6, 7, and 8).

For *instances *of cumulative-constitutively organized material entities holds:

1. Every object aggregate entity must have some object entity as its proper part that belongs to the same CO granularity level as its name giving object.

2. Not every object entity that belongs to the same CO granularity level as the name giving object of an object aggregate or a fiat object part must be proper part of a corresponding object aggregate or fiat object part entity.

3. Every fiat object part entity must have some object entity as its proper part that belongs to a lower CO granularity level than its name giving object.

#### 6.2.4 Two Basic Spatial Granularity Perspectives

**Figure 9:**
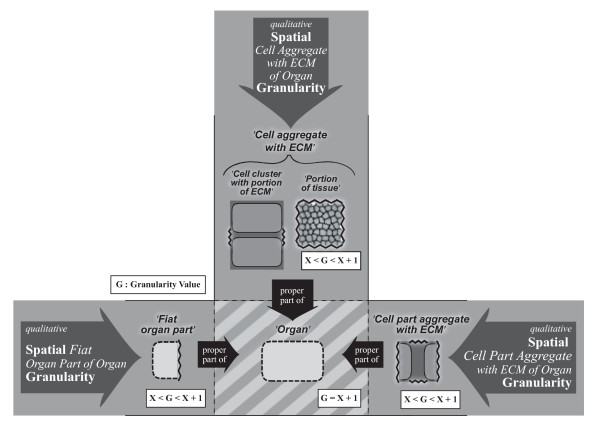
**Basic Spatial Granularity Perspectives**. A combination of three particular qualitative *spatial fiat object part of object granularity perspectives*. Each perspective is defined in reference to a combination of proper parthood and either the defining properties of specific subtypes of 'object' (in the perspectives depicted it is 'organ'), all of which belong to the same compositional object granularity level, or the defining properties of their respective 'fiat object part' parts (in the perspectives depicted these are 'cell aggregate with ECM', 'fiat organ part', and 'cell part aggregate with ECM'). Each basic spatial granularity perspective contains only two levels: a lower level, to which subtypes of 'fiat object part' belong (e.g., 'cell aggregate with ECM', 'fiat organ part', or 'cell part aggregate with ECM'), and a higher level, to which subtypes of 'object' belong (e.g., 'organ'). Notice that the three perspectives shown overlap with one another at their 'organ' levels - a particular organ can belong to all three perspectives at the same time. **ECM**: extracellular matrix.

With the CO perspective and its different granularity levels in the background, one can differentiate two different types of basic spatial granularity perspectives. A ***spatial fiat object part of object granularity perspective***, which is based on a proper parthood relation between a specific subtype of 'object' and its corresponding parts, which at their turn must represent regional parts and thus specific subtypes of 'fiat object part' (for examples see Fig. 9). Furthermore, although of less relevance for cumulative-constitutively organized entities, a ***spatial fiat object part of object aggregate granularity perspective***, which is based on a proper parthood relation between a specific subtype of 'object aggregate' and specific subtypes of 'fiat object part' as its corresponding parts.

The *granulation criterion *of these two different types of particular basic spatial granularity perspectives thus combines the proper parthood property with the defining properties of either a specific subtype of 'object' or 'object aggregate' that is directly or indirectly assigned to a particular granularity level of the CO perspective or of the specific corresponding subtype of 'fiat object part'.

i)   'fiat object part'   *is-direct-proper-part-of*   'object';

      'object'   *has-direct-proper-part*   'fiat object part'.

And of limited relevance:

ii)   'fiat object part'   *is-direct-proper-part-of*   'object aggregate';

      'object aggregate'   *has-direct-proper-part*   'fiat object part'.

As a consequence, only objects that belong to the same CO granularity level, or their respective object aggregates, and their corresponding fiat object part parts belong to the same particular spatial granularity perspective. From this follows that for each type of basic spatial granularity there exist as many different particular perspectives as there are different levels of CO granularity. The particular spatial perspectives are demarcated from one another according to the defining properties of the respective 'object' or 'object aggregate' subtypes, which at their turn must belong either directly or indirectly to the same CO granularity level. Put differently, each particular basic spatial granularity perspective is assigned to a particular CO granularity level.

Each particular basic spatial granularity perspective has granulation of the non-scale dependent single-relation-type *granularity type *(*nrG *[32]).

Each consists of only two granularity levels, which are defined and demarcated according to the properties of the 'object' or 'object aggregate' subtypes versus those of the 'fiat object part' subtypes that the perspective contains. The granularity level to which the 'fiat object part' subtypes belong represents the lower level, and entities belonging to this lower level are related to entities belonging to the higher level through proper-part-of relations.

Therefore, despite the arbitrariness that is connected to all partitions involving regional parts, these parts all have a proper parthood relation to their respective object or object aggregate entity in common. As a consequence, no matter how one actually partitions a given object or object aggregate entity into regional parts (e.g., see Fig. 9), these parts will always represent proper parts of the given object or object aggregate entity. They will always belong to the lower granularity level of its respective basic spatial perspective and the corresponding object or object aggregate entity to the higher one.

By distinguishing different subtypes of 'fiat object part' one can differentiate further subtypes of spatial granularity perspectives, which, however, would go beyond the scope of this paper (for examples see Fig. 7, 8, and 9).

For *instances *of cumulative-constitutively organized material entities holds:

1. Not every fiat object part entity must be proper part of an object or object aggregate entity that is directly or indirectly assigned to the adjacent higher CO granularity level (e.g., not every portion of tissue is proper part of some organ).

2. Every fiat object part entity must be proper part of some object entity that belongs to the same CO granularity level as its name giving object entity.

3. Every object or object aggregate has at least two fiat object part entities as its proper part.

4. For a given granulation, every fiat object part entity must be proper part of the same maximum object entity.

#### 6.2.5 Two Basic Scale Dependent Resolution Granularity Perspectives

**Figure 10:**
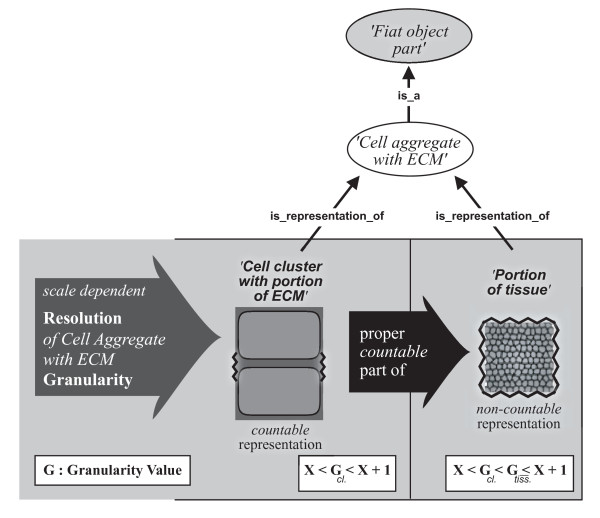
**Resolution Granularity Perspective**. A particular quantitative scale dependent *resolution of object aggregate with fiat object part granularity perspective*. Each resolution perspective is defined in reference to a combination of a proper countable parthood relation with a is-representation-of relation and the defining properties of all subtypes of 'object aggregate' or 'object aggregate with fiat object part' that belong to the same *compositional object aggregate of object granularity perspective *or the same *spatial fiat object part of object granularity perspective *respectively (depicted is the resolution perspective of cell aggregate with ECM, a particular *resolution of object aggregate with fiat object part granularity perspective*). Each resolution granularity perspective contains only two levels: a lower level, to which subtypes of 'object cluster' or 'object cluster with portion of substance' (e.g., 'cell cluster with portion of ECM') belong that represent countable representations of the corresponding subtypes of 'object aggregate' or 'object aggregate with fiat object part' (e.g., 'cell aggregate with ECM') respectively, and a higher level, to which subtypes of 'portion of substance' or 'portion of granular mixture of substance *X *and *Y*' (e.g., 'portion of tissue') belong that represent the non-countable representations of the same object aggregate or object aggregate with fiat object part. **ECM**: extracellular matrix.

Besides the various qualitative granularity perspectives presented above one can differentiate several quantitative (i.e. scale dependent) structural granularity perspectives in reference to different subtypes of 'object aggregate' or a specific type of fiat object part, 'object aggregate with fiat object part'. A ***resolution of object aggregate granularity perspective***, which is of limited relevance for cumulative-constitutively organized entities, and a ***resolution of object aggregate with fiat object part granularity perspective***. Each type of resolution granularity perspective is based on a countable/non-countable representation-of relation of an object aggregate or of an object aggregate with fiat object part: (i) the countable 'object cluster' or 'object cluster with portion of substance' representation and (ii) the non-countable 'portion of substance' or 'portion of granular mixture of substance *X *and *Y*' representation respectively (for an example see Fig. 10). The countable/non-countable representation-of relation represents a subtype of the proper-part-of relation [32]. The *granulation criterion *of a particular resolution perspective thus combines a proper parthood relation with a is-representation-of relation and the defining properties of all subtypes of 'object aggregate' or 'object aggregate with fiat object part' that belong to the same *compositional object of object aggregate granularity perspective *or the same *basic spatial fiat object part of object granularity perspective *respectively:

i)   'object cluster with portion of substance'      *is-countable-proper-part-of*   'portion of granular mixture of substance X and Y';

      'portion of granular mixture of substance X and Y'      *has-countable-proper-part*   'object cluster with portion of substance'.

And of limited relevance:

ii)   'object cluster'      *is-countable-proper-part-of*   'portion of substance';

      'portion of substance'   *has-countable-proper-part*   'object cluster'.

Particular resolution perspectives are demarcated from one another according to the defining properties of the respective subtypes of 'object aggregate' or 'object aggregate with fiat object part' that must belong to the same *compositional object aggregate of object granularity perspective *or the same *spatial fiat object part of object granularity perspective*. In other words, each particular resolution perspective is assigned to a particular compositional or spatial granularity perspective.

Each particular resolution perspective has granulation of the scale dependent grain-size-according-to-resolution *granularity type *(*sgrG *[32]). It uses a specific form of proper parthood relation as its *granulation relation *and is in this respect compatible to the other spatio-structural granularity perspectives proposed above.

Each particular resolution perspective consists of only two granularity levels. They are defined according to the is-a-representation-of property and the defining properties of its corresponding subtype of 'object aggregate' or 'object aggregate with fiat object part'. They are demarcated from one another according to the property of being countable or non-countable respectively. The granularity level to which the countable representations belong represents the lower level, and entities belonging to this lower level are related to entities belonging to the higher level through is-countable-proper-part-of relations.

### 6.3 Integrating Different Spatio-Structural Granularity Perspectives within a General Spatio-Structural Granularity Framework

The different types of granularity perspectives proposed above all meet the requirements put forward by Keet's general theory of granularity [32]:

• All instances or types in any of the proposed granularity levels have at least one aspect in common, which is the granulation criterion.

• Each proposed granularity perspective contains at least two granularity levels.

• Adjacent fine and coarser-grained granularity levels of any here proposed granularity perspective are related to one another in two ways: (i) Instances or types belonging to adjacent levels are related to one another according to the *granulation relation*. (ii) The adjacent levels themselves are related to one another according to a binary proper parthood relation *RL *[32]. *RL *is identical for all levels and all granularity perspectives [32]. This clear distinction between relating levels on the one hand and relating their contents on the other hand is necessary since the latter, the *granulation relation*, requires a more precise specification than *RL *[32].

• Each level is contained in exactly one particular granularity perspective. However, any particular instance or type may belong to more than one perspective, but in each perspective it must belong only to one granularity level. In the here proposed granularity perspectives it especially applies to objects, which can belong to many different perspectives at the same time.

The question is how the different structural granularity perspectives can be combined within a common general spatio-structural granularity framework. How do the different perspectives relate to each other, how do the levels from different perspectives? How do the perspectives relate to their overarching spatio-structural granularity framework?

The integration of different granularity perspectives within a common granularity framework can be achieved by relating levels of distinct perspectives through relating their perspectives [32]. The binary relation *RP *between two distinct perspectives is *irreflexive *and *symmetric *[32]. On the basis of this *RP *relation, according to Keet [32], one can identify two strategies for directly linking levels of different perspectives: (i) using overcrossing levels with mereology and (ii) using chaining levels with *RL *and *RP*.

Two levels of different perspectives *overcross *if they, although representing different levels of distinct perspectives, share at least some of their contents, which thus *overlap*. And if levels of distinct perspectives overcross, the perspectives themselves overcross as well [32]. This is the case for all the granularity perspectives here proposed. They all share content in at least one of their levels with another perspective. Together, they form a continuous chain or network of overcrossing perspectives - one can traverse all here proposed perspectives through overlapping contents. All compositional perspectives discussed above, for instance, overcross with the *compositional object perspective *or the *object aggregate of object aggregate perspective*, of which the latter is of limited relevance for cumulative-constitutively organized entities (see Fig. 6, 7, and 8). The two basic spatial granularity perspectives discussed above each share, for example, content of their higher granularity level with content from one level of the *compositional object granularity perspective *or the less relevant *compositional object aggregate of object aggregate granularity perspective *(see Fig. 7, 8, and 9). The basic spatial granularity perspectives as well as the additional compositional perspectives thus all overcross with the *compositional object granularity perspective*.

The scale dependent *resolution of object aggregate with fiat object part perspective *and the less relevant *resolution of object aggregate perspective*, on the other hand, do not overcross with the *compositional object granularity perspective*. However, both of their granularity levels share content with other spatial and compositional granularity perspectives that contain subtypes of 'fiat object part' or 'object aggregate' in one of their granularity levels. Therefore they overcross with various additional compositional and basic spatial granularity perspectives (for examples see Fig. 6, 7, 8, and 9).

Within the here proposed general integrated spatio-structural granularity framework, the *compositional object granularity perspective *takes in a central role, since its levels provide the content that is shared with many of the framework's other granularity perspectives. It thus provides the backbone to the entire spatio-structural granularity framework.

Now, all that is still required in order to integrate granularity levels, granularity perspectives, and the subject domain into a common granularity framework, is another relation which relates granularity levels with their perspective and perspectives with their subject domain. According to Keet [32], this is a *transitive *and *acyclic *(i.e. an object *X *does not have a path to itself) binary containment relation *RE*, which is a subtype of the proper parthood relation (for an overview of the different relations between the various components of Keet's general theory of granularity see Figure 3.1, p. 48 in [32]).

### 6.4 Assigning Structural Granularity Values to Spatio-Structural Granularity Levels

An interesting question that arises when looking at the general integrated spatio-structural granularity framework presented here is whether it is possible to order all granularity levels across different perspectives according to an overarching single general spatio-structural granularity perspective. Such a perspective would have to be very general and thus rather abstract in order to cover all the different perspectives by overcrossing with all their levels, which seems to be impossible on the type level. However, when considering the structural organization of a particular cumulative-constitutively organized material entity, it is possible to assign a *structural granularity value *to each level of granularity of each perspective of the framework. In order to be able to do so, one has to utilize the overcross-relations between different perspectives within the framework. Overcrossing levels must share the same structural granularity value. Here, again, does the *compositional object granularity perspective *function as a backbone. One can assign a fix natural number to each of its levels. The lowest level receives the number one. Each subsequent level receives the next higher natural number, so that adjacent fine and coarser-grained levels possess the structural granularity value *X *and *X*+1, with *X *being an element of the natural numbers (see Fig. 5). Now, all granularity levels of other granularity perspectives that overcross the *compositional object granularity perspective *share the same fix structural granularity value with the corresponding overcrossing level. This applies to the finer grained level of most of the additional compositional perspectives and the higher level of the basic *spatial fiat object part of object granularity perspective *(see Fig. 6 and 9).

Due to the *arbitrariness *of regional parts, however, the non-overcrossing levels of both the additional compositional granularity perspectives and the basic spatial granularity perspectives cannot possess fix structural granularity values. Instead, only *relative structural granularity values *can be assigned. These relative values are assigned in relation to the fix values of the overcrossing levels. The relative values are in between the fix values and do not represent natural numbers anymore. For instance the coarser-grained 'cell aggregate with ECM' level of the *compositional cell of cell aggregate with ECM perspective *possesses a structural granularity value that is higher than the value of the finer grained 'cell' level, but lower than the adjacent coarser-grained corresponding 'organ' level of the backbone *compositional object granularity perspective *(Fig. 6 and 7). Moreover, the coarser-grained 'cell aggregate with ECM' level of the *compositional cell of cell aggregate with ECM perspective *and the finer-grained 'cell aggregate with ECM' level of the basic *spatial cell aggregate with ECM of organ perspective *share the same relative structural granularity value since their contents overlap (Fig. 7). But then, the relative structural granularity values of the two levels of for example the *resolution of cell aggregate with ECM granularity perspective *both fall within the range of the relative structural granularity value of the corresponding 'cell aggregate with ECM' level of both the *spatial cell aggregate with ECM of organ perspective *and the *compositional cell of cell aggregate with ECM perspective*. Within this value range, however, the fine-grained 'cell cluster with ECM' representation can receive a relative structural granularity value that is equal to or less than the value of the corresponding coarser-grained 'portion of tissue' representation (Fig. 7 and 10) (see also discussion in the next paragraph).

The resulting order of structural granularity values with fix natural numbers linked to different types of objects on the one hand and relative values linked to different types of object aggregates and fiat object parts on the other hand seems to reflect an idea it has in common with the Foundational Model of Anatomy (FMA [11]). The FMA distinguishes *salient levels of structural organization *(i.e. *Biological macromolecule *<*Cell *<*Portion of tissue *<*Organ *<*Organ system *<*Cardinal body part *<*Body*), which are defined in reference to *units of structural organization *(i.e. objects and object aggregates) from *transitional *or *intermediate levels *(i.e. *Cardinal cell part *<*Cardinal tissue part *<*Cardinal organ part *<*Organ system subdivision *<*Subdivision of cardinal body part*), which are defined in reference to *subdivisions *or *cardinal parts *of objects (i.e. regional parts, fiat object parts). The *transitive *or *intermediate *nature that Rosse and Mejino [11] assert for granularity levels containing regional parts is reflected in the relative structural granularity values in the granularity framework proposed here. However, the FMA framework does not differentiate the different types of granularity perspectives involved and thus suffers from the problems discussed further above.

### 6.5 Additional Spatial Granularity Perspectives

One can introduce another type of spatial granularity perspective in addition to those proposed above, a ***spatial fiat object part of fiat object part granularity perspective***. Each fiat object part entity can itself be partitioned into its regional parts, which at their turn can be partitioned again. These partitions are based on proper parthood relations between fiat object parts and form what could be called second (or even higher) order regional parts: they are regional parts of regional parts.

The *granulation criterion *of this additional type of spatial granularity perspective combines the proper parthood property with the defining properties of the involved subtypes of 'fiat object part':

   'fiat object part'      *is-proper-part-of*               'fiat object part';

   'fiat object part'      *has-direct-proper-part*      'fiat object part'.

**Figure 11:**
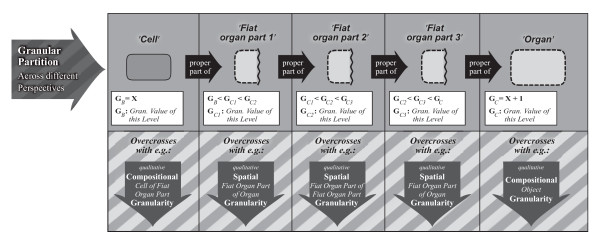
**Example of how Structural Granularity Values are assigned to a given Partition**. Example for a particular granular partition of an organ into its fiat organ part and cell parts. Shown is a chain of proper parthood relations, starting from a particular cell, that is part of a specific fiat organ part 1, which at its turn is part of a fiat organ part 2 that is part of a fiat organ part 3, which is part of an organ. The structural granularity values of the respective object entities (i.e. cell and organ) can be inferred using the fix natural number values from the compositional object granularity perspective: value X for the cell entity and value (X+1) for the organ entity. The structural granularity values of the different fiat organ part entities must be within the range of X and (X+1), as the overcrossing spatial granularity perspectives imply. With the *finer-grained *relation ' < ', the respective value interval 'X to (X+1)' can be further confined for each particular fiat organ part entity in the way it is shown in this figure.

This additional spatial granularity perspective can also be integrated into the general spatio-structural granularity framework: The additional spatial perspective overcrosses with other spatial and compositional perspectives of the framework. The additional perspective concerns only fiat object parts and not objects. As a consequence, the respective levels do not overcross with any level of the backbone compositional object granularity perspective, each of which possesses a fix natural number structural granularity value. Instead, they overcross with levels of other perspectives, which at their turn only possess relative structural granularity values. Since these values represent real number intervals, they can accommodate an infinite number of smaller real number intervals for structural granularity values. This allows to assign structural granularity value intervals to each level of the *spatial fiat object part of fiat object part granularity perspective *that is within the range of relative structural granularity values of the granularity levels with which the perspective overcrosses (Fig. 11). The only thing that is required is an additional *finer-grained*/*coarser-grained *relation that further confines the structural granularity value interval for the granularity levels of the *spatial fiat object part of fiat object part perspectives *and that demarcates all granularity levels from one another that belong to the same broader structural granularity value interval.

The problem with the additional spatial granularity perspective is that a given object entity can be partitioned in many different ways into various different regional parts, some of which will even overlap one another. These regional parts themselves could be partitioned into various different regional parts as well. While all these different regional parts *necessarily *represent proper parts of their corresponding object entity and thus necessarily belong to some of the above mentioned spatial granularity perspectives, they are *not necessarily *proper parts of one another. For instance, not all possible fiat object parts of a given object necessarily belong to the same granular partition - some fiat object parts will overlap with one another and only some will be proper parts of one another. As a consequence, theoretically for any given object there are an infinite number of different additional *spatial fiat object part of fiat object part granularity perspectives *possible that contain 'fiat object part' levels, although only one of them could be realized when having to cut the object into actual fiat object parts. Therefore, the application of *spatial fiat object part of fiat object part granularity perspectives *should be restricted to instances (i.e. particulars) and not to types (i.e. universals).

### 6.6 Scale Dependent Size Granularity Perspectives

One can also granulate all types of material entities solely according to a specific type of scale. Such a quantitative ***size granularity perspective ***would not rest on any type of parthood relation. Instead, it is based on some *larger-than*/*smaller-than *relation in combination with a particular type of scale (i.e. all types of properties that are projectible on a metrical measure, e.g. max. width, surface, volume, weight, max. lifespan). Arbitrary measure-thresholds demarcate and order its various granularity levels. Such granularity perspectives are usually only applicable to the instance level - at least in biology, the variability and diversity among instances of the same type does not allow such a perspective to be applied to the type level. Instead of structural granularity values, respective granularity levels would have different *scale granularity values *assigned.

## 7 Conclusions

The most important consequence from accepting the existence of cumulative-constitutively organized material entities is that all their parts of one granularity level *do not always *exhaustively sum to the whole in a granularity tree - not all entities belonging to one level of granularity always form parts of entities of the next higher level of granularity (contradicting [36]). This has far reaching consequences for any theory of granularity, which have been discussed in detail above. Another problem of most commonly used granularity schemes is that they commit to a granularity framework that does not allow the integration of different granularity perspectives within a common framework.

The problem that Kumar et al.'s [13] granularity scheme for human anatomy had to face regarding their basic granularity principles, with its levels of 'cardinal body part' and 'organ system' violating their second (i.e. *grains in a given level are parts of grains in the next higher level*) and their fifth principle (i.e. *grains in a given level must be smaller in size than those entities on the next higher level of which they are parts*), can be avoided. The problem does not occur when following the principles mentioned in the previous section, and when precisely distinguishing different granularity perspectives and integrating them to an overall spatio-structural granularity framework, instead of forcing them into a single perspective. Transferred to the general integrated spatio-structural granularity framework presented here, a human respiratory system would be an organ cluster with portion of tissue and, due to its cumulative constitutive organization, a fiat body part. The human head and chest, on the other hand, are fiat body parts as well. In their granularity scheme for human anatomy, however, Kumar et al. [13] treat the latter (i.e. 'Cardinal body part') as the finer grained level of granularity and the former (i.e. 'Organ system') as the adjacent coarser grained level, although both are actually fiat body parts. Transferred to the here presented integrated framework, their structural granularity values occupy the same value range.

The only granularity perspective, in which both a cardinal body part and an organ system could belong to adjacent levels of granularity is a *spatial fiat object part of fiat object part granularity perspective*. This perspective, however, can be only applied reasonably to the granulation of individual material entities - i.e. to instances, but not to types.

However, since both the respiratory system, as well as the head and the chest, are proper parts of a human body, and since both belong to granularity levels that share the same structural granularity value range, there might be some organisms in which *some *cardinal body part is proper part of *some *organ system (and vice versa). But this is not necessarily the case for *every *cardinal body part and every organ system, and most certainly not for every organism. Therefore, the respective types cannot belong to adjacent granularity levels of a common *type *granularity perspective.

With the here presented integrated granularity framework, any particular spatio-structural granular partition of any given material entity can be modeled and to each of its particular regional and constitutive parts an unambiguous structural granularity value can be assigned, which consistently increases from each part to whole relation. In other words, any particular proper parthood relation between two material entities can be assigned to one of the here proposed granularity perspectives. As a consequence, the rather artificial distinction between gross and granular parthood suggested by Rector et al. [69], which differentiates between a *gross parthood *for parthood relations between entities of the same granular level, and a *granular parthood *for parthood relations between entities belonging to different levels of granularity, is not required in the here presented granularity framework.

Moreover, the framework does not require an a priori ordering of parts - all that is required are the proper parthood relations. The assignment of the structural granularity values can be inferred on the basis of the granularity framework, using the *compositional object perspective *as the backbone that provides the fix natural number values. In a next step, the various other compositional, spatial, and resolution-dependent perspectives are accordingly joined with the *compositional object perspective *and with one another via overcrossing granularity levels, contributing the relative real number structural granularity intervals. This is possible because in the integrated framework, (i) all granularity perspectives are connected to one another through overcrossing granularity levels, together forming an integrated whole with no perspective being cut off from the other perspectives; (ii) every granularity perspective is based on a proper parthood relation. As a consequence, any spatio-structural granularity partition can be modeled. Any given spatio-structural granular partition with already assigned structural granularity values can be granulated even further (e.g., through adding levels to the *spatial fiat object part of fiat object part granularity perspective*), without causing inconsistencies within the granularity framework and without having to change any of the already assigned structural granularity values.

The here presented framework provides a spatio-structural granularity framework not only for biological material entities, but for all domain reference ontologies that model cumulative-constitutively organized material entities. With its multi-perspectives approach and the possibility to include even granularity perspectives that are based on taxonomic inclusion (i.e. is-a relations), it allows querying an ontology stored in a database at one's desired different levels of detail: The contents of a database can be organized according to diverse granularity perspectives, which at their turn provide different *views *on its content (i.e. data, knowledge), each organized into different levels of detail. This not only allows for detailed and sophisticated searches within the database, enhancement of its information and knowledge management, granular zooming in and out, and abstracting away of irrelevant details while focusing on the level of detail relevant for one's specific information needs [32]. It also can be very valuable for making inferences utilizing computer reasoning (e.g. [70,71]). It would even allow the use of different taxonomies. For example a taxonomy of morphological types exclusively based on spatio-structural properties and organized through is-a relations, alongside a taxonomy of functional types exclusively based on dispositions and organized through is-functionally-a relations. Each taxonomy could follow the single inheritance rule, and all relevant relations of taxonomic inclusion still could be modeled in the same ontology through different taxonomic granularity perspectives.

Granularity, in general, is best understood - at least when used in combination with ontologies and databases - as an additional layer on top of an existing domain reference or a terminology-based application ontology. It functions like a meta-ontology, which organizes the respective ontology according to some general granularity framework, thereby organizing the properties and relations present in the underlying ontology according to different granularity perspectives. Whereas the here presented general integrated spatio-structural granularity framework is restricted to granularity perspectives that are based on relative location (i.e. spatial partitions) and structural composition (compositional partitions), both of which rest on structural proper parthood relations, it does not necessarily have to adhere to these restrictions. It could, for instance, be integrated with a granularity framework that is based on causal dispositions (i.e. functional parthood relations), on temporal partitions, on developmental properties and relations, on genealogical relations, or on evolutionary origin. This would facilitate the often called for integration of life-science data, which demands a single framework in which all different disciplines and their respective data types can be involved (see also [12,13]).

## Authors' contributions

LV developed the here presented framework and wrote the manuscript.
